# Modelling exposure heterogeneity and density dependence in onchocerciasis using a novel individual-based transmission model, EPIONCHO-IBM: Implications for elimination and data needs

**DOI:** 10.1371/journal.pntd.0007557

**Published:** 2019-12-05

**Authors:** Jonathan I. D. Hamley, Philip Milton, Martin Walker, Maria-Gloria Basáñez

**Affiliations:** 1 London Centre for Neglected Tropical Disease Research (LCNTDR), Department of Infectious Disease Epidemiology, School of Public Health, Faculty of Medicine (St Mary’s campus), Imperial College London, London, United Kingdom; 2 MRC Centre for Global Infectious Disease Analysis, Department of Infectious Disease Epidemiology, School of Public Health, Faculty of Medicine (St Mary’s campus), Imperial College London, London, United Kingdom; 3 London Centre for Neglected Tropical Disease Research (LCNTDR), Department of Pathobiology and Population Sciences, Royal Veterinary College, University of London, Hatfield, Untied Kingdom; University of Zurich, SWITZERLAND

## Abstract

**Background:**

Density dependence in helminth establishment and heterogeneity in exposure to infection are known to drive resilience to interventions based on mass drug administration (MDA). However, the interaction between these processes is poorly understood. We developed a novel individual-based model for onchocerciasis transmission, EPIONCHO-IBM, which accounts for both processes. We fit the model to pre-intervention epidemiological data and explore parasite dynamics during MDA with ivermectin.

**Methodology/Principal findings:**

Density dependence and heterogeneity in exposure to blackfly (vector) bites were estimated by fitting the model to matched pre-intervention microfilarial prevalence, microfilarial intensity and vector biting rate data from savannah areas of Cameroon and Côte d’Ivoire/Burkina Faso using Latin hypercube sampling. Transmission dynamics during 25 years of annual and biannual ivermectin MDA were investigated. Density dependence in parasite establishment within humans was estimated for different levels of (fixed) exposure heterogeneity to understand how parametric uncertainty may influence treatment dynamics. Stronger overdispersion in exposure to blackfly bites results in the estimation of stronger density-dependent parasite establishment within humans, consequently increasing resilience to MDA. For all levels of exposure heterogeneity tested, the model predicts a departure from the functional forms for density dependence assumed in the deterministic version of the model.

**Conclusions/Significance:**

This is the first, stochastic model of onchocerciasis, that accounts for and estimates density-dependent parasite establishment in humans alongside exposure heterogeneity. Capturing the interaction between these processes is fundamental to our understanding of resilience to MDA interventions. Given that uncertainty in these processes results in very different treatment dynamics, collecting data on exposure heterogeneity would be essential for improving model predictions during MDA. We discuss possible ways in which such data may be collected as well as the importance of better understanding the effects of immunological responses on establishing parasites prior to and during ivermectin treatment.

## Author summary

Onchocerciasis, caused by the helminth parasite *Onchocerca volvulus*, is transmitted via the bites of *Simulium* blackflies. The World Health Organization has proposed onchocerciasis elimination in African countries by 2020/2025. Processes regulating parasite abundance in the lifecycle of helminths are known to influence the endemic prevalence in mathematical models. For example, when transmission intensity is low, a high proportion of incoming parasites may establish within a human host, whilst the opposite may be true when transmission intensity is high, possibly due to immunological processes. These processes may interact with exposure as some people are bitten more than others and receive more parasites. Therefore, regulatory processes that depend on parasite density and inter-individual variation in exposure play a central role in the ability of transmission to bounce back following mass drug administration. The former, because they may increase the success of parasite establishment as treatment progresses; the latter, because a few highly infected individuals may maintain transmission. We developed an individual-based model for onchocerciasis transmission and show that the interaction between these two processes impacts treatment outcomes. We highlight the need to obtain data on exposure to vector bites and to understand how immunological processes potentially regulating parasite establishment change under treatment.

## Introduction

The World Health Organization (WHO)’s roadmap on neglected tropical diseases [1] has earmarked onchocerciasis for elimination by 2020 in selected African countries, and the Joint Action Forum (JAF) of the WHO African Programme for Onchocerciasis Control (APOC) proposed elimination in 80% of endemic countries by 2025 [2]. As onchocerciasis programmes based on mass drug administration (MDA) of ivermectin transition from morbidity control to parasite elimination [3], the usefulness of mathematical models will rest on our ability to identify and understand processes that may make parasite populations resilient to MDA and able to persist at low prevalence [4].

**Fig 1:**
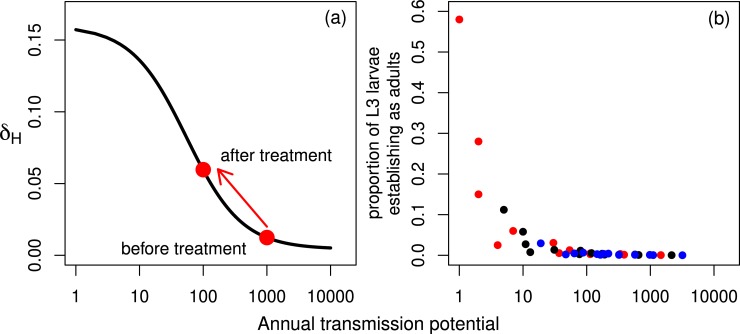
Density dependence in the establishment of adult *Onchocerca volvulus*. **(a) Density-dependent establishment of worms in the human host (**$\delta_{H}=\left[\frac{{\delta_{H0}+\delta }_{H\infty }c_{H}ATP}{1+c_{H}ATP}\right]$, **as described by [18] and using the parameterisation in [6]).** The red points represent hypothetical values of δ_*H*_ before and after treatment. *δ*_*H*0_ is the proportion of L3 larvae developing to the adult stage within the human host, per bite, when *ATP* → 0; *δ*_*H*∞_ is the proportion of L3 larvae developing to the adult stage within the human host, per bite, when *ATP*(*t*) → ∞ and *c*_*H*_ is the severity of transmission intensity-dependent parasite establishment. **(b) The model and data in [9] are used to produce the equivalent of Dietz’s function [18], for the mean ATP values (black points), and their upper and lower confidence intervals (blue and red respectively) as reported in [9].** The *O*. *volvulus* nodule (onchocercoma) establishment rate per village is given by $NER=\frac{1}{Z}\sum_{i=1}^{n}{NER}_{i}\frac{z_{a}}{n_{a}},$ where *NER*_*i*_ = *k*_*i*_/*a*_*i*_; *k*_*i*_ is the number of nodules palpated in individual *i* at age *a*_*i*_; *Z* is the number of people across all villages entering the analysis for which nodulectomy (and paired ATP) data were available for 14 villages in the Onchocerciasis Control Programme in West Africa (OCP) database; *n* is the population size of the village under consideration; *n*_*a*_ is the number of people aged *a*, and *z*_*a*_ is the number of people aged *a* across all villages. The incidence of adult worms (parasite establishment rate, *PER*) in a village is then, $PER=\frac{m}{p}\;NER$, where *p* is the proportion of nodules accessible to palpation, and *m* is the average number of adult female worms in a nodule. We set *m* (which was multiplied by 2 to find the total number of adult worms, assuming a balanced sex ratio) and *p* to values proposed by [34].

Density-dependent processes acting on various parts of parasite lifecycles are well recognised as an important aspect of helminth transmission dynamics, stabilising parasite populations and contributing to their resilience—their capacity to recover—during (and after) control interventions [5,6,7,8,9]. Positive or facilitative density dependencies (e.g. the mating probability in dioecious, separate sexes species) limit transmission at low parasite population densities and create so-called transmission breakpoints [10]. Negative or constraining density-dependent processes limit transmission at high parasite population densities and, as they are relaxed during intervention, enhance transmission at low population densities [6,7,11]. It follows that density-dependent parasite establishment may have important implications for the resilience of onchocerciasis to interventions [12, 13, 14]. Since parasites are typically overdispersed among hosts (i.e. the variance is substantially greater than the mean worm load), the severity of density dependence differs among individuals and, therefore, the net population-level effect is altered by the degree of parasite overdispersion [10,15]. A description of the biological motivation for accounting for a variety of density-dependent processes in onchocerciasis transmission models can be found in [6], [9], [16] and [17]. There are two representations of density dependence in the establishment of the parasite life stage in humans. The first was introduced by Dietz [18] and was later used in the deterministic precursor of the EPIONCHO model (see [22] for a review) and is also adapted for use (for individual human hosts) in this paper. The proportion of parasites that establish in humans is considered to be a function of the annual transmission potential (*ATP* = *L*3 × *ABR*, the number of L3 larvae potentially received per person per year, where *L*3 is the mean number of L3 larvae in the fly population and *ABR* is the annual biting rate (the average number of bites received per person per year)). This decreasing proportion of establishing parasites with increasing ATP has implications for parasite resilience during mass drug administration (Fig 1A). The red points indicate how the proportion of establishing L3 larvae may increase after treatment as the ATP declines. This increase in the proportion of establishing parasites constrains the ability of treatment to reduce transmission because—although the number of parasites in the fly population is reduced—a higher proportion of these parasites establish. Duerr *et al*. (2006) [9] presented evidence for density-dependent establishment using individual-level data. The equivalent output of Dietz’s function can be reproduced using data from the 14 villages with paired nodulectomy and ATP information in the OCP database, and the model in [9] (Fig 1B). Similarly, to Fig 1A, we see that the proportion of establishing adult parasites decreases as the ATP increases.

In *Onchocerca volvulus*, density-dependent processes affect the proportion of microfilariae (mf, the progeny of adult worms) establishing in blackfly (*Simulium* spp.) vectors [16], the survival of infected blackflies [17], and the establishment of juvenile larvae as adult worms in humans [18,19,20]. A large amount of work has considered the impact of these density dependencies on the transmission dynamics of onchocerciasis using population-based deterministic models [6,21,22,23]. However, these approaches have not permitted investigation of the interactive effects of density dependence with heterogeneity in individuals’ exposure to blackfly bites.

Individuals may differ in their exposure to blackfly bites due to their attractiveness to flies [24], occupation [25,26] or age and sex [21,27]. Variation in exposure to vector bites has received theoretical and empirical attention in the context of lymphatic filariasis [28,29], schistosomiasis [30] and malaria [31,32] but is less well studied for onchocerciasis (although see [33]). In lymphatic filariasis, high levels of exposure heterogeneity are associated with increased resilience to MDA, allowing parasite persistence at low prevalence [28]. Moreover, it has been shown that identical prevalence values can be produced by different combinations of vector to host ratios (indicative of population *average* exposure) and levels of heterogeneity; high exposure heterogeneity reduces parasite prevalence [28] and therefore, a higher vector to host ratio is required to achieve a given prevalence. Thus, it has been suggested that prevalence alone should not determine expectations on intervention success [28]; a population with high prevalence and high exposure heterogeneity will be more resilient to treatment than a population with the same prevalence, but lower heterogeneity in exposure.

Here, we consider how the interaction between overdispersion in exposure to vector bites and density-dependent establishment of *O*. *volvulus* in human hosts affects the parasite’s population dynamics. We present a novel individual-based stochastic onchocerciasis transmission model, EPIONCHO-IBM (an analogue of the population-based deterministic EPIONCHO model (see [22] for a recent review)), which we parameterise using historical pre-intervention epidemiological data from savannah settings in Africa to capture the interaction between exposure heterogeneity and density-dependent parasite establishment within humans. We use the model to explore the impact of these interactive processes on *O*. *volvulus* population dynamics during MDA with ivermectin. We discuss how uncertainty in key parameters influences our projections and highlight data requirements for improving the accuracy of model-based predictions. In addition to individual-based models (IBMs) being suited to modelling heterogeneity in exposure (and potentially also in susceptibility) to infection, they are particularly useful in context of public health questions (for example, allowing the consideration of sensitivity and specificity to diagnostic tests; accounting for screen and treat protocols, among others). A broader aim of this work is, therefore, to develop a tool with which a wide range of questions (not within the scope of this paper) may be answered that is not possible with its deterministic precursor (EPIONCHO [22]).

## Methods

There are three principal methodological components of this work: i) development of a novel individual-based onchocerciasis transmission model (EPIONCHO-IBM); ii) parameterisation of heterogeneity in exposure to blackfly bites and density-dependent establishment of worms within humans, and iii) simulations to investigate the role of these processes and their interactions on the dynamics of *O*. *volvulus* during MDA with ivermectin.

### EPIONCHO-IBM

EPIONCHO-IBM is a stochastic, individual-based analogue of a previously developed population-based model, EPIONCHO (see [22] for a recent review and [23] for the latest refinements to the deterministic version). EPIONCHO-IBM follows each human in a closed population, keeping track of the number of infecting adult *O*. *volvulus* (of each sex and reproductive status) and microfilariae. The presence of both male and female worms is required for the production of microfilariae, assuming a completely polygamous mating system [10, 34]. The model accounts for age- and sex-dependent exposure of humans to blackfly bites, as in the deterministic version of the model [21], whilst additionally incorporating individual-level variation in exposure. An individual-specific exposure factor is assigned at birth and is drawn from a gamma distribution,

$$E_{\left(i\right)}∼gamma(k_{E},\beta_{E})$$ (1)

where *k*_*E*_ and *β*_*E*_ are the shape and rate parameters, respectively. We assume always that *k*_*E*_ = *β*_*E*_, such that the mean exposure in the population is unity, permitting the ABR (the average number of bites per person per year), to be distributed among the host population.

### Parasite life-history traits

We assume senescence in parasite longevity and fecundity based on existing data [35]. Mortality rates of adult worms and microfilariae are assumed to increase with age, according to a Weibull model fitted, in the case of microfilariae, to data presented by [36] (S1 Text, Text A, Formal description of EPIONCHO-IBM, Fig A). Following [37], parasite fecundity decreases with age (Fig B in S1 Text). Figures showing the dependency of parasite life history traits (mortality and fecundity) on age, and the resulting fit of the model to temporal (declining) trends of community microfilarial load (CMFL, as defined in [38]) since the inception of vector control in Burkina Faso villages of the Onchocerciasis Control Programme in West Africa (OCP), are included in S1 Text (Fig C).

### Parasite population regulation

Density-dependent processes are assumed to act on three stages of the *O*. *volvulus* lifecycle, namely: establishment of larvae within the vector; parasite-induced mortality of the vector, and establishment of adult worms within the human. Herein, reference to ‘density dependence parameters’ refers to the density dependence in the establishment of the parasite in humans unless otherwise specified, and the parameters determining other density-dependent processes remain fixed [23]. After accounting for individual-level variation, as well as age- and sex-specific exposure, the density-dependent establishment of adult worms in individual *i* is given by,

$$\Pi_{H(i)}\left[ATP\left(t-\tau_{H}\right),\Omega_{T}\left(a_{\left(i\right)}-\tau_{H}\right)\right]=\left[\frac{\delta_{H0}+\delta_{H\infty }c_{H}ATP\left(t-\tau_{H}\right)\Omega_{T}\left(a_{\left(i\right)}-\tau_{H}\right)}{1+c_{H}ATP\left(t-\tau_{H}\right)\Omega_{T}\left(a_{\left(i\right)}-\tau_{H}\right)}\right]$$ (2)

where *τ_H_* is the delay between L3 larvae entering the host and establishing as adult worms to account for the duration of development into L4 stages and L5 (juvenile adults), *a_(i)_* is the age of individual *i*, Ω_*T*_(*a_(i)_*) is the total exposure for individual *i*, *δ*_*H*0_ is the proportion of L3 larvae developing to the adult stage within the human host per bite when *ATP(t)* → 0, *δ_H∞_* is the proportion of L3 larvae developing to the adult stage within the human host per bite when *ATP(t)* → ∞ and *C_H_* is the severity of transmission intensity-dependent parasite establishment within humans.

The model accounts for a latent period in the development of the parasite in the vector by including L1, L2 and L3 stages [23, 39], based on data for African (Cameroon) settings [40]. The dynamics of the parasite within the vector are modelled deterministically at a fly population level, as in the deterministic EPIONCHO model [22, 23]. This population-based modelling of the vector population is also found in other individual-based models for vector borne diseases [28, 41]. Treatment with ivermectin is assumed to have a large but finite microfilaricidal effect which decreases with time since treatment (following the dynamics presented in [42]). Ivermectin temporarily sterilises some female worms while making others permanently infertile [43]. A complete description and mathematical definition of the model, together with parameters values, is given in S1 Text (Text A: Formal description of EPIONCHO-IBM, Tables A to H, Figs A to D).

### Parasitological data and model parameterisation

The degree of exposure heterogeneity and density dependence in parasite establishment in humans were estimated by fitting the model simultaneously to pre-intervention microfilarial prevalence and intensity (mean number of microfilariae per mg of skin) data and their corresponding annual biting rate (ABR, the number of bites per person per year) from savannah settings in [44, 45] (Northern Cameroon) and [46] (Burkina Faso and Côte d’Ivoire). Although the model explicitly considers hypothetical individuals (which then allow the calculation of microfilarial prevalence and mean microfilarial infection intensity in the population), no individual-based data on exposure were used for its parameterisation as these data are not yet available at suitable scales. Instead, population-based epidemiological data, which were collected from different locations and timepoints, were used to parameterise the model regarding the processes of interest (i.e. exposure and density dependence within humans). For other processes, the model was parameterised based on epidemiological setting-independent data (e.g. density dependence within blackflies; effects of ivermectin on parasite life stages, etc.), as described in [22, 23] and S1 Text.

Parameter sets for density dependence were generated using Latin hypercube sampling (LHS, *n* = 100), with each set simulated for a range of *k*_*E*_ values (0.2, 0.3, 0.4). (S1 Text, Text B: Uncertainty and sensitivity analysis using the Latin hypercube parameter sets, Fig E, illustrates the results for *k*_*E*_ = 0.3 in the form of box-and-whiskers plots for microfilarial prevalence and intensity.) The estimation of the density dependence parameters separately for each value of *k*_*E*_ allows the simulation of MDA for each level of exposure heterogeneity, and consequently exploration of how uncertainty in this parameter may influence the outcome of intervention programmes. The fit produced by each value of *k*_*E*_ (with the corresponding density dependence parameters estimated for that value of *k*_*E*_) can then be compared.

We calculated the sum of squared residuals (the discrepancy between the modelled microfilarial prevalence and intensity and the observed data) as a measure of the goodness-of-fit of each parameter set. Residuals were normalised between 0 and 1 to allow the prevalence and intensity to influence the fitting equally. We calculated the partial rank correlation coefficients for *δ*_*H*0_, *δ*_*H*∞_ and *c*_*H*_ (following [47]) to quantify how each parameter influenced the pre-intervention model predictions (Fig F in S1 Text).

The microfilarial prevalence and intensity data used to fit the model [44, 45, 46] were complemented with additional data from [9] (OCP, microfilarial prevalence only) and other epidemiological settings with simuliid species without cibarial armatures and vector competence characteristics similar to those of *Siumulium damnosum* sensu stricto (s.s.)/*S*. *sirbanum* [7], namely Amazonian focus communities with transmission by *S*. *guianense* sensu lato (s.l.) [48], and Ecuadorean communities with transmission by *S*. *exiguum* s.l. [49, 50] to validate the model fit. For microfilarial prevalence data, binomial 95% confidence intervals (95% CIs) were calculated using the Clopper-Pearson method [51]. Individual host microfilarial intensity data were only available for [44] and these were used for calculating 95% CIs around the arithmetic means using bootstrapping [52].

### Population dynamics during mass drug administration (MDA) with ivermectin

We used the best fit parameter sets (for each value of *k*_*E*_) which gave the lowest sum of squared residuals to project the dynamics of microfilarial prevalence (from two skin snips, assuming that a Holth corneoscleral punch-derived skin snip weighs, on average, 2 mg [22]) during MDA with ivermectin. Since ivermectin has been distributed in savannah areas of northern Cameroon since the late 1980’s we generated predictions for annual (yearly) treatment for 25 years. For comparative purposes, we also simulated biannual (6-monthly) treatment for the same programme duration. We made predictions for populations with baseline microfilarial prevalence values ≈ 30% (hypoendemic), 50% (mesoendemic), 60% and 70% (hyperendemic). The choice of baseline conditions was motivated by the frequency of those that were observed in the OCP prior to the commencement of control [53]. We assume that 1% of the population are non-compliant (they never receive treatment), and that the mean treatment probability in any treatment round is 0.8 (i.e. there is 80% therapeutic coverage of the total population). An overview of how compliance structure influences resilience to treatment in a generalized setting can be found in [54].

## Results

### Endemic microfilarial prevalence and intensity

**Fig 2:**
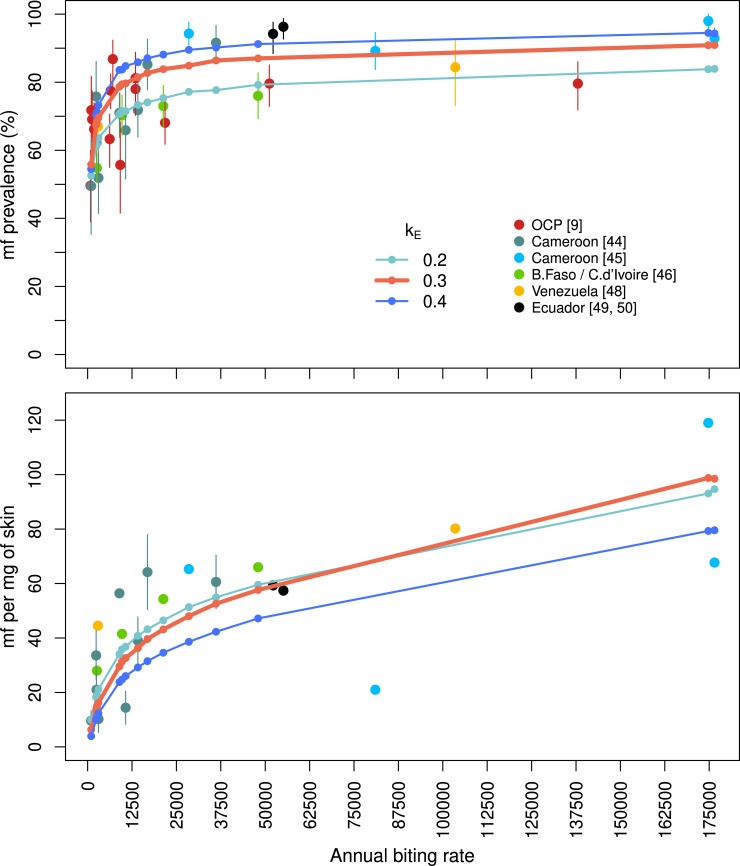
Pre-intervention *Onchocerca volvulus* microfilarial prevalence and intensity vs. the annual biting rate of simuliid vectors. The predicted microfilarial prevalence (percent) and microfilarial intensity (mean no. of microfilariae, mf, per mg of skin) (from 2 skin snips) for the annual vector biting rates reported in the combined epidemiological dataset (i.e. fitting and validation data, solid colour circles), using the estimated parameters, are represented by solid lines. The best-fit parameter values of the density-dependent parasite establishment within humans were *δ*_*H*0_ = 0.186, *δ*_*H∞*_ = 0.003 and *c*_*H*_ = 0.005 for exposure heterogeneity parameter *k*_E_ = 0.3 (thick red line); *δ*_*H*0_ = 0.385, *δ*_*H*∞_ = 0.003, *c*_*H*_ = 0.008 for *k*_E_ = 0.2 (thin light blue line), and *δ*_*H*0_ = 0.118, *δ*_*H*∞_ = 0.002, *c*_*H*_ = 0.004 for *k*_E_ = 0.4 (thin dark blue line). The EPIONCHO-IBM predictions are based on a host population size of 500 and 100 runs for 80 years to reach endemic equilibrium (human demography was simulated to equilibrium before simulating the epidemiology to equilibrium). The error bars are binomial (Clopper-Pearson) 95% confidence intervals for prevalence and bootstrapped 95% CIs for intensity (for which the raw individual microfilarial intensity data were available [44]). The main vectors in each setting are *Simulium damnosum* s.s./*S*. *sirbanum* (African savannah in Benin, Burkina Faso, Cameroon, Côte d’Ivoire, Ghana, Guinea, Mali, Togo); *S*. *guianense* s.l. (Amazonian focus in southern Venezuela) and *S*. *exiguum* s.l. (Cayapas focus in Ecuador), all species are without armed cibaria and have vector competence features similar to those of *S*. *damnosum* s.s. [7]. Fitting data are from [44, 45, 46]; validation data are from [9, 48, 49, 50]. The prevalence and intensity data points for an annual biting rate of 81,000 were excluded from the fitting of the model as according to [45] a large number of flies were nulliparous.

Fig 2 shows the relationship between pre-control microfilarial prevalence, arithmetic mean microfilarial intensity and ABR, using the parameters obtained by LHS that achieved the minimum squared residuals compared with the observed data from [44, 45, 46] for each value of *k*_*E*_. A value of *k*_*E*_ = 0.3 and the associated density dependence parameters (*δ*_*H*0_ = 0.186, *δ*_*H*∞_ = 0.003, *c*_*H*_ = 0.005) gave the lowest squared residuals.

When using *k*_*E*_ = 0.3, EPIONCHO-IBM captures well not only the pre-intervention observed microfilarial prevalence and intensity across the biting rates used for comparing the model fit to data, but also the other data sources used for validation (i.e. excluded from the least squares calculation) [9, 48, 49, 50]. A value of *k*_*E*_ = 0.2 predicted well the microfilarial intensity but tended to under predict microfilarial prevalence; conversely, a value of *k*_*E*_ = 0.4 underpredicted intensity but was better able to capture microfilarial prevalence across all ABRs than *k*_*E*_ = 0.2. Fig 2 shows that model predictions generated by values of *k*_*E*_ = 0.2, 0.3 and 0.4 encompass most of the prevalence and intensity data points in the range of ABR values explored (1,000 to 176,500). Figures showing microfilarial prevalence and intensity for all parameter sets from the Latin hypercube sample (for *k*_*E*_ = 0.3), as well as the influence of individual density dependence parameters on (endemic equilibrium) model outputs for a range of ABR values, are shown in S1 Text, Text B (Fig E and Fig F, respectively).

### Threshold biting rate

**Fig 3:**
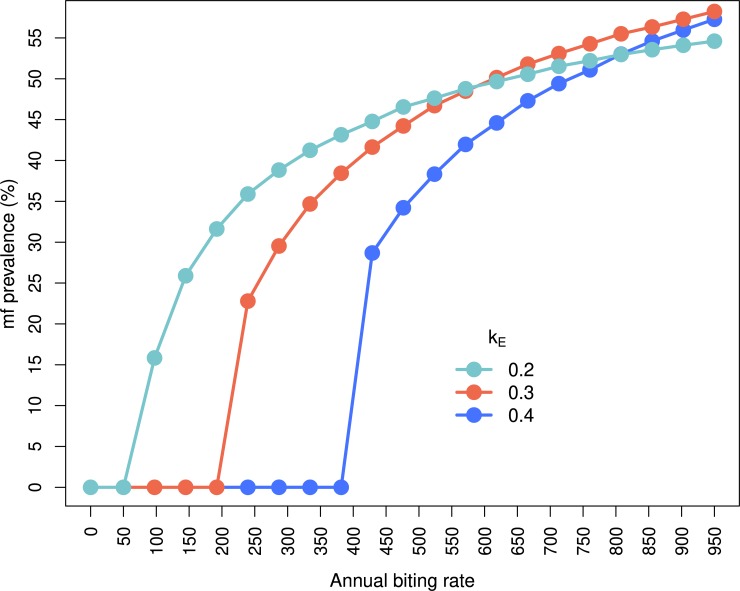
Threshold biting rates for each endemic microfilarial prevalence varying the exposure heterogeneity parameter *k*_E_ and associated best-fit parameters of the density-dependent parasite establishment within humans. Endemic prevalence values of 0 indicate annual biting rates (number of bites per person per year) that are below the so-called threshold biting rate (TBR) for transmission (indicating that the basic reproduction number *R*_0_ <1). The EPIONCHO-IBM predictions are based on a host population size of 500, 1000 runs and for 80 years to endemic equilibrium, assuming 63% of blood meals are taken on humans [55]. Density dependence parameter values for each value of *k*_E_ are as in Fig 2 TBR values are 97, 239 and 429 for *k*_E_ = 0.2, 0.3 and 0.4, respectively.

Increasing the level of exposure heterogeneity (i.e. decreasing the value of *k*_*E*_) decreases the threshold biting rate (TBR, the minimal ABR necessary for endemic onchocerciasis, i.e. for which the basic reproduction number of the parasite, *R*_0_, is equal to 1 [16]), giving TBR values ranging from approximately 97 for *k*_*E*_ = 0.2 to 429 for *k*_*E*_ = 0.4, assuming that the proportion of bloodmeals taken on humans is 0.63 for *S*. *damnosum* s.s./*S*. *sirbanum* [50] (Fig 3).

### Population and individual-level parasite establishment within humans

**Fig 4:**
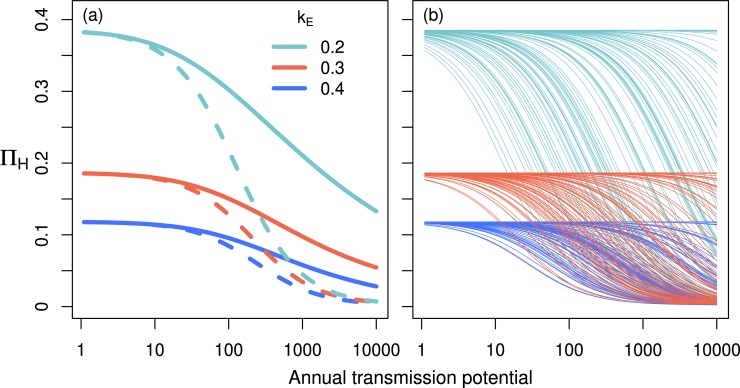
The EPIONCHO-IBM density-dependent parasite establishment within-humans using the best-fit estimates of the parameters *δ*_*H*0_, *δ*_*H*∞_ and *c*_*h*_ for each value of *k*_E_. In panel (a), solid lines show the population mean density dependence with age-, sex- and individual-specific heterogeneity in exposure to vector bites. Dashed lines show the density-dependent establishment function without heterogeneity in exposure using the same parameters. Panel (b) shows the density dependence for 100 individuals in the stochastic setting (each line is for one individual) for each value of *k*_E_. The mean of these lines (for each exposure heterogeneity value) gives the solids lines in panel (a). Density dependence parameter values for each value of *k*_E_ are as in Fig 2. Note that the y-axis label has been abbreviated for presentational purposes; specifically for panel (b) this is *П_H(i)_*, in panel (a), we represent the value given by taking the mean output of this function for all individuals in the population with *П_H_* (solid lines), and represent the function without exposure heterogeneity using *П_H_* also (dashed lines).

We see also that the population mean density dependence in the stochastic model developed here departs from that found in the simpler deterministic framework without exposure heterogeneity (when using the estimated density dependence parameters from the stochastic model) (Fig 4A). In the stochastic setting, density-dependent establishment of the parasite in an individual depends on their exposure to fly bites (Eq 2), which is determined by a gamma-distributed individual level exposure, as well as age- and sex-dependent exposure. This creates a distribution of Π_*H*_ curves in the host population (Fig 4B), which can vary depending on *k*_*E*_ (as well as on the host sex ratio and age distribution). Since (for the *k*_*E*_ values tested), most individuals in a population have low exposure (and therefore weak density-dependent establishment relative to the annual transmission potential), there is a higher mean proportion of establishing parasites in the human population for higher annual transmission potentials than in the deterministic model.

### Dynamics of microfilarial prevalence under ivermectin treatment

**Fig 5:**
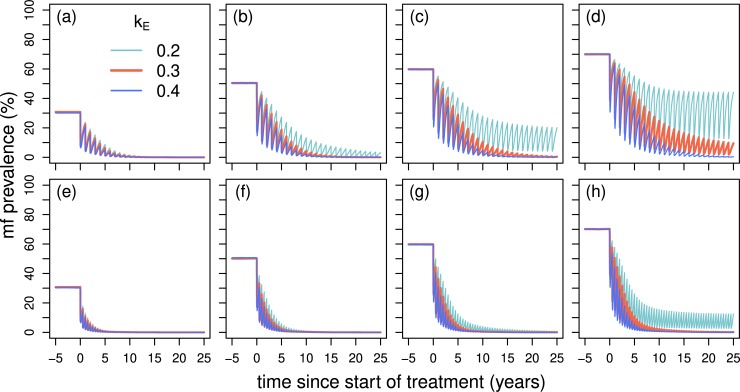
**Microfilarial prevalence dynamics during 25 years of annual (a–d) and biannual (e–h) mass drug administration (MDA) with ivermectin for various levels of endemicity, heterogeneity in exposure to vector bites (measured by *k***_**E**_**) and the associated density-dependent establishment parameters.** The baseline microfilarial prevalence (30%, 50%, 60%, 70%, indicative of hypo-, meso-, hyper-, and high hyperendemicity, respectively) is modelled by increasing the annual biting rate. At each treatment round, 80% of the population receive treatment, excluding children aged <5 years who are ineligible to receive ivermectin. We assume 1% of the total population are systematic non-adherers (i.e. they never take treatment during the programme duration). The EPIONCHO-IBM predictions are based on a host population size of 500, 1000 runs and with 80 years to reach endemic equilibrium before initiating MDA. Parameters for the thick red lines (*k*_E_ = 0.3), thin light blue lines (*k*_E_ = 0.2) and thin dark blue lines (*k*_E_ = 0.4) are as in Fig 2.

Treatment dynamics indicate that resilience to MDA is markedly higher for *k*_*E*_ = 0.2 than for *k*_*E*_ = 0.3 and 0.4. This difference between the different levels of exposure heterogeneity tended to increase as the pre-intervention microfilarial prevalence increased but decreased with an increase in treatment frequency (Fig 5). Although this difference in resilience between the different values of *k*_*E*_ is in part due to the resulting exposure heterogeneity, it is also associated with different estimated strengths of density dependence (Fig 4). More heterogenous exposure leads to the estimation of stronger density dependence, principally due to a higher proportion of parasites establishing as ATP decreases. Since increasing exposure heterogeneity (reducing *k*_*E*_) reduces the microfilarial prevalence, a higher proportion of parasites establishing (than when exposure is less overdispersed) is required to reach a given observed prevalence and intensity. Simulations assuming either a fixed level of exposure heterogeneity, and different strengths of density dependence, or a fixed level of density dependence and different levels of exposure heterogeneity are shown in S1 Text, Text B: Uncertainty and sensitivity analysis using the Latin hypercube parameter sets, Figs G and H.

## Discussion

We have developed a novel stochastic individual-based onchocerciasis transmission model, EPIONCHO-IBM, based on the deterministic population-based analogue, EPIONCHO. EPIONCHO-IBM captures explicitly the interactive effects of heterogeneity among humans in exposure to blackfly vector bites and density-dependent processes that operate fundamentally at the individual host level and that are key determinants of the resilience of parasite populations to intervention. Modelling this interaction is, therefore, essential for predicting the likely impact of interventions on parasite population dynamics and ultimately the feasibility of elimination. This is the first study to explore this effect in the context of onchocerciasis using epidemiological data to parameterise and validate these interactive population processes. In doing so, we demonstrate that data collection on exposure heterogeneity may make an important contribution to reducing uncertainty when modelling parasite dynamics during MDA.

The range of exposure heterogeneity levels explored (*k*_*E*_ = 0.2 to 0.4) fits to the variation in pre-control microfilarial prevalence and intensity with ABR, encompassing most of the data points collated (including fitting and validation datasets), with *k*_*E*_ = 0.3 providing the best overall fit. A value of *k*_*E*_ = 0.2 tended to underestimate prevalence but fitted better low prevalence values, whilst *k*_*E*_ = 0.4 tended to overestimate prevalence but fitted better high prevalence. The results suggest that exposure heterogeneity may decrease as the annual biting rate increases (i.e. *k*_*E*_ increases with increasing ABR).

This decrease in heterogeneity with increasing ABR may represent that, in a small population of blackflies, the physical limit on how many times a fly can bite constrains the number of people that may be bitten. This would result in a high heterogeneity in exposure to bites. Conversely, a large population of flies is capable of biting many people, leading to lower levels of heterogeneity in exposure to bites. If such a relationship between exposure heterogeneity and ABR were supported by other data sources and incorporated into the model, it may have substantive implications for the predicted population dynamics during MDA. In particular, the degree of heterogeneity in exposure would be less in highly endemic settings with high prevalence and high ABRs. Consequently, the resilience to intervention in these settings would be reduced and the impact of MDA enhanced. The projected parasite population dynamics during treatment with ivermectin would likely be somewhat more closely aligned with the corresponding deterministic predictions of EPIONCHO, which incorporates a functional relationship between the degree of adult worm overdispersion and ABR [23]. The assumption of a fixed level of exposure heterogeneity (either across the wide range of annual biting rates collated, as in Fig 2, or for single modelled endemic communities, as in Fig 5) is unavoidable due to data availability. More likely, there will be a distribution of *k*_*E*_ values depending on the specific ecological and sociological context of a geographical area. Even if such a distribution allowed *k*_*E*_ to vary between 0.2 and 0.4, we might expect substantial spatial variation in population responses to treatment for a given baseline microfilarial prevalence.

The threshold biting rate decreased with increasing levels of exposure heterogeneity which is consistent with similar work on lymphatic filariasis [28]. Furthermore, the threshold biting rate for the best fit parameters was similar to previous deterministic versions of the model assuming the same proportion of blood meals taken on humans (380 in [6], 300 in [19]), but lower than a different model based on the same data (730 [20]).

A key distinction between EPIONCHO-IBM and its deterministic counterpart is that in the former, overdispersion is generated mechanistically via heterogeneity in exposure and density dependence operating at the level of the individual host. In EPIONCHO, the effects of parasite overdispersion on the severity of density dependence are modelled empirically [15, 21, 22, 23]. More generally, worm overdispersion in the host population is an input in deterministic population-based models, and an output of individual-based stochastic models (calculated by fitting a negative binomial distribution to predicted worm burdens), depending on exposure heterogeneity and potentially density dependent processes. This is a crucial difference and implies individual-based models are not simply approximations of their deterministic counterparts. How density-dependent processes influence the relationship between exposure heterogeneity and parasite overdispersion (of adult worms and microfilariae) in EPIONCHO-IBM is an important question which remains to be addressed and will be presented elsewhere.

The phenomenological nature of density-dependent establishment of the parasite in the human host is an important limitation of this work. The use of population-level data to simultaneously estimate both density dependence and exposure heterogeneity allows the parameters involved to counteract, reducing identifiability and diminishing mechanistic interpretation. Since direct estimation of density-dependent parasite establishment within humans is not feasible experimentally (with the only available observational study on parasite establishment rates being that of [9]), data collection on heterogeneity in exposure to fly bites will be an important step in better resolving density dependence. This has been discussed previously [26] but available data remain limited. Since blackflies have very specific environmental requirements, with breeding sites varying in distance to human settlements, the estimation of individual-level variation in exposure to bites poses a substantial challenge.

Heterogeneity in exposure to mosquito bites has been estimated by comparing DNA extracted from (indoor resting) mosquito bloodmeals with DNA from individuals living in study households [32]. Since *Anopheles* mosquitoes breed in standing water, which may be littered throughout villages, this is a viable method for finding mosquitoes which have fed on study participants. In the context of onchocerciasis, this process may be impractical because blackflies do not bite and rest indoors, and outdoor resting sites are very difficult to find, so studies investigating bloodmeal origin rely on host-dependent and host-independent sampling methods [55]. The former (human landing catches) would require a large number of participants to find enough flies which that could be matched to the individuals on which they previously fed, and the latter (using oviposition traps in nearby breeding sites) may not sample sufficient flies which would have fed on the residents of the nearby communities [55]. Irvine *et al*. [29] have suggested there may be spatial variation in exposure heterogeneity to mosquito bites, adding an additional layer of complexity and uncertainty in obtaining generalizable estimates. Perhaps more suitable for estimating exposure to blackfly bites is the method which has recently been applied in an empirical study of *Leishmania infantum* transmission among dogs in Brazil [56]. Levels of (IgG) antibody responses to sandfly saliva were found to vary with the intensity of transmission, declining after periods of low transmission, as well as to vary greatly between dogs, correlating with the intensity of transmission experienced by individual dogs. Studies such as these require the development of anti-saliva antibody assays, which are currently lacking in simuliids (partly because their colonisation in the lab is very difficult). If these assays could be developed in the context of onchocerciasis, based initially on titres of antibody responses to crude blackfly salivary antigens, they could be used in population samples across all age and sex groups and may provide useful data for estimating heterogeneity in individual-level exposure. Such a study may also provide data to increase the accuracy of assumptions regarding age- and sex-dependent exposure. Currently this is estimated by fitting models to data on age- and sex-specific profiles of microfilarial prevalence and intensity [21]. However, these modelled age and sex exposure profiles are determined not by exposure to vectors and parasite infective stages alone, but also by processes involving parasite development and parasite fecundity within the host, as they rely on (downstream) microfilarial data. Reducing the uncertainty in age- and sex-dependent exposure patterns is particularly important for optimal selection of age (and potentially sex) groups for (serological) assessment of exposure to parasite antigens in foci thought to be nearing elimination [57]. Ideally, assays for anti-blackfly saliva could be combined with assays for exposure to *O*. *volvulus* to investigate both exposure to vector bites and parasite antigens (e.g. Ov16 [58]).

In addition to exposure, susceptibility to infection may also vary between individual hosts. Work on schistosomiasis has shown that there is marked variability in susceptibility to infection [59, 60], and that accounting for both exposure and susceptibility can better capture *Schistosoma* transmission to snails than accounting for exposure alone [61]. It is possible, that what we term exposure heterogeneity, does also account for individual variation in susceptibility, given the way it is estimated. However, as there is a lack of data on individual heterogeneity in exposure and susceptibility to infection, making this distinction explicit is unlikely to lead to better parameterisation or improved accuracy of the model. In addition, since density-dependent establishment of adult worms might represent immunological processes (which interact with exposure at the individual level in the model), it is not necessarily clear how variation in susceptibility (which is likely determined by genetic and immunological processes) should be accounted for. Therefore, although we do not account for variation in susceptibility explicitly, the proportion of incoming parasites which establish in an individual depends on their exposure, implying a relationship between exposure and susceptibility.

The density-dependent within-human parasite establishment function used in EPIONCHO and EPIONCHO-IBM, derived from the work in [18], is a phenomenological representation of a possible immune-mediated response to infective (L3) larvae driven by the intensity of exposure to these parasite stages. In areas of low ATP (or in individuals with little exposure), low levels of exposure to L3 larvae may be responsible for a poor development of protective immune responses, leading to high parasite establishment rates. Conversely, in areas of high ATP (or in individuals highly exposed), stronger immune responses against incoming worms may decrease parasite establishment rates. The operation of anti-L3 responses in putative immunes living in areas of hyperendemic onchocerciasis has been shown by [62, 63]. In the *Teladorsagia circumcincta*–Scottish Blackface sheep parasite–host system, priming of the immune system early in the season by exposure to (ingestion of) infective L3 larvae in pasture, reduces parasite establishment and growth and, therefore, faecal egg counts in a density-dependent fashion, not unlike our own parasite establishment function (see Figs 2 and 3 of [64]).

Density-dependent processes may also (or instead) act on parasite fecundity [65]. Here we assume for simplicity, that one worm of each sex is required for reproduction, i.e. that one male worm is sufficient for all adult female worms to be fertilised and produce mf [10, 34], and that increasing female worm density does not reduce the per capita fecundity rate [19]. However, if in addition to density-dependent parasite establishment, the fecundity rate was to decrease as the density of adult parasites increases, we might expect additional resilience during treatment, since the fecundity rate per worm would increase as MDA reduces the number of parasites in the population. Of interest, previous work indicates that, when investigated separately, density-dependent fecundity contributes less to the rate of bounce back following treatment than density-dependent adult worm establishment [8].

The EPIONCHO-IBM projections indicate that there is a disproportionate increase in resilience with increasing pre-intervention prevalence for *k*_E_ = 0.2. That is, as pre-intervention prevalence increases, particularly from low hyperendemic (microfilarial prevalence ≈ 60%) to high hyperendemic (microfilarial prevalence ≈ 70%) settings, the *O*. *volvulus* population becomes disproportionally more resilient to MDA. This can be partly understood in the context of the strongly nonlinear prevalence vs. ABR relationship, in which prevalence begins to saturate at ABR values exceeding 7,000 (daily biting rates > 20/person, ~70% mf prevalence). This phenomenon would also be found for lower levels of exposure heterogeneity, as the prevalence vs. ABR relationship begins to saturate, albeit at higher ABRs. Another contributory factor to resilience to MDA-based interventions in our model is that our parasite establishment function is not affected by the potential interaction between microfilaricidal treatment and the immune response. Therefore, as treatment progresses and the ATP declines, the parasite establishment rate inevitably increases. However, ivermectin-facilitated immunity in onchocerciasis has been reported [66, 67]. Human immunological studies have demonstrated that filarial parasites induce a state of hypo-responsiveness in the host that is associated with the presence of circulating mf (patent infection), and *O*. *volvulus* is no exception [68]. The reversal of this mf-associated immune-suppression, following clearance of skin mf due to ivermectin treatment, may contribute to controlling *O*. *volvulus* infection [66]. In the *O*. *ochengi*–cattle system, animals treated with ivermectin and exposed to blackfly bites under natural transmission conditions did not develop patent infections whilst treated but recovered their susceptibility and acquired infection (at rates higher than untreated counterparts) once treatment stopped [69]. Similar observations have been made in a large pharmaco-epidemiological study of *Dirofilaria immitis* in dogs [70]. A better understanding of how ivermectin-facilitated immunity could impact the establishment of incoming worms in human onchocerciasis would greatly improve the modelling of parasite establishment rates during the implementation and after cessation of MDA programmes.

The microsimulation model for onchocerciasis ONCHOSIM [22, 33, 41, 71], which also uses a gamma distribution to model individual-level variation in exposure, uses parameter *k*_E_ as 1 or 3.5, giving substantially less exposure heterogeneity than in EPIONCHO-IBM (*k*_E_ = 0.3). It has been shown that EPIONCHO (the deterministic analogue of EPIONCHO-IBM) predicts more resilience to intervention than ONCHOSIM under a range of treatment scenarios (using *k*_E_ = 3.5 for ONCHOSIM) [13, 14]. Although our treatment dynamics were simulated under high (and likely unrealistic) levels of therapeutic coverage and adherence, the programme duration (25 years) was motivated by that in the Vina valley of northern Cameroon (from which the data used to estimate the parameters investigated in this paper originated [44, 45]). In Cameroon, there was no interruption of transmission after 15 [72, 73], 17 [74], 18 [75] or 25 years [76] of annual ivermectin MDA in some communities. Although this may be due to a number of factors including lower than reported coverage, decreased ivermectin efficacy [77], movement of infected individuals between foci and spatial variation in exposure heterogeneity, it may indicate that the more pessimistic predictions of EPIONCHO-IBM for hyperendemic populations, are not necessarily out of touch with observed trends. It is noteworthy that EPIONCHO-IBM can mimic the behaviour of ONCHOSIM (i.e. less pessimistic treatment dynamics) by removing density-dependent parasite establishment within humans (absent in ONCHOSIM) and increasing *k*_E_. This process—and its interaction with heterogeneity in exposure—is a critical determinant of the different population dynamics predicted by the two models (to be formally discussed elsewhere).

EPIONCHO-IBM has a wider scope of application than its population-based predecessor, albeit at the cost of reduced tractability (a general drawback of many individual-based stochastic models). A particular advantage is the capacity to model individual-based interventions. For example, individuals co-infected with *O*. *volvulus* and *Loa Loa* are at risk of severe adverse events (e.g. encephalitis) following treatment with ivermectin if harbouring heavy loiasis microfilaraemia [78]. Consequently “test and treat” (testing for *O*. *volvulus*) or “test and not treat” (testing for *L*. *loa*) strategies have been proposed and trialled for control in areas co-endemic with onchocerciasis and loiasis [79]. EPIONCHO-IBM, as an individual-based model, can simulate such strategies, incorporating performance features (i.e. sensitivity, specificity) of a variety of diagnostics and detailed information on observed screening, coverage and adherence patterns among demographic groups. It follows that co-infection dynamics may also be modelled, although the within-host interaction between *O*. *volvulus* and *L*. *loa* and helminth species more generally is poorly understood (but see [80]).

## Conclusions

In conclusion, we have developed a novel individual-based stochastic onchocerciasis transmission model, EPIONCHO-IBM, based on the well-established deterministic analogue, EPIONCHO. We have used EPIONCHO-IBM to better understand how density-dependent processes—in particular the density-dependent establishment of newly acquired *O*. *volvulus* parasites—and heterogeneity in individual human exposure shape both the relationships between microfilarial prevalence, microfilarial intensity and ABR, and the resilience of onchocerciasis to MDA with ivermectin. In future, EPIONCHO-IBM will be used to model the control and elimination of onchocerciasis using current and alternative interventions, including the use of anti-*Wolbachia* therapies [81], moxidectin [82], ground-based vector control [83] and new macrofilaricidal therapies [84]. Our work also highlights the importance and uncertainty in the key and interactive population processes of density dependence and heterogeneity in exposure to blackfly vectors. Novel approaches for generating data on exposure heterogeneity and anti-L3 immunity during and after the cessation of ivermectin MDA programmes would be particularly valuable in helping to resolve outstanding uncertainty on their relative importance to the population dynamics of *O*. *volvulus*. Combined with more statistically advanced approaches for estimating the parameters of individual-based models [85], and fitting such models to longitudinal epidemiological trends, such data would greatly enhance the reliability and accuracy of onchocerciasis modelling projections.

## Supporting information

S1 Text Detailed description of EPIONCHO-IBM and additional results. A) Formal description of EPIONCHO-IBM. B) Uncertainty and sensitivity analyses using the Latin hypercube parameter sets.
(DOCX)

## References

[1] World Health Organization. Accelerating work to overcome the global impact of neglected tropical diseases. A roadmap for implementation. Geneva: World Health Organization 2012; WHO/HTM/NTD/PCT/2012.1. http://www.who.int/neglected_diseases/NTD_RoadMap_2012_Fullversion.pdf. Accessed 28 May 2019.

[2] African Programme for Onchocerciasis Control (APOC). Eighteenth Session of the Joint Action Forum. Bujumbura, Burundi, 11–13 December 2012. Final Communique. http://www.who.int/apoc/about/structure/jaf/Final_Communique_JAF_18_English_final_with_annexes.pdf. Accessed 28 May 2019.

[3] Colebunders R, Basáñez MG, Siling K, Post RJ, Rotsaert A, Mmbando B, et al From river blindness control to elimination: bridge over troubled water. Infect Dis Poverty. 2018;7(1):21 10.1186/s40249-018-0406-7 29587844 PMC5872540

[4] Duerr HP, Dietz K, Eichner M. Determinants of the eradicability of filarial infections: a conceptual approach. Trends Parasitol. 2005; 21(2):88–96. 10.1016/j.pt.2004.11.011 15664532

[5] Dietz K. Density-dependence in parasite transmission dynamics. Parasitol Today. 1988;4(4):91–97. 10.1016/0169-4758(88)90034-8 15463054

[6] Basáñez MG, Collins RC, Porter CH, Little MP, Brandling-Bennett D. Transmission intensity and the patterns of *Onchocerca volvulus* infection in human communities. Am J Trop Med Hyg. 2002;67(6):669–679. 10.4269/ajtmh.2002.67.669 12518860

[7] Basáñez MG, Churcher TS, Grillet ME. *Onchocerca-Simulium* interactions and the population and evolutionary biology of *Onchocerca volvulus*. Adv Parasitol. 2009;68: 263–313. 10.1016/S0065-308X(08)00611-8 19289198

[8] Churcher TS, Filipe JAN, Basáñez MG. Density dependence and the control of helminth parasites. J Anim Ecol. 2006;75(6):1313–1320. 10.1111/j.1365-2656.2006.01154.x 17032363

[9] Duerr HP, Leary CC, Eichner M. High infection rates at low transmission potentials in West African onchocerciasis. Int J Parasitol. 2006;36(13):1367–1372. 10.1016/j.ijpara.2006.08.001 16979644

[10] May RM. Togetherness among schistosomes: its effects on the dynamics of the infection. Math Biosci. 1977;35:301–343.

[11] Anderson RM, May RM. Helminth infections of humans: mathematical models, population dynamics, and control. Adv Parasitol. 1985;24:1–101. 10.1016/s0065-308x(08)60561-8 3904343

[12] Walker M, Stolk WA, Dixon MA, Bottomley C, Diawara L, Traoré MO, et al Modelling the elimination of river blindness using long-term epidemiological and programmatic data from Mali and Senegal. Epidemics. 2017;18:4–15. 10.1016/j.epidem.2017.02.005 28279455 PMC5340858

[13] Stolk WA, Walker M, Coffeng LE, Basánez MG, de Vlas SJ. Required duration of mass ivermectin treatment for onchocerciasis elimination in Africa: a comparative modelling analysis. Parasit Vectors. 2015;8:552 10.1186/s13071-015-1159-9 26489937 PMC4618738

[14] Verver S, Walker M, Kim YE, Fobi G, Tekle AH, Zouré HGM, et al How can onchocerciasis elimination in Africa be accelerated? Modeling the impact of increased ivermectin treatment frequency and complementary vector control. Clin Infect Dis. 2018; 66(Suppl 4):S267–S274. 10.1093/cid/cix1137 29860291 PMC5982715

[15] Churcher TS, Ferguson NM, Basáñez MG. Density dependence and overdispersion in the transmission of helminth parasites. Parasitology. 2005; 131(1):121–132. 16038403 10.1017/s0031182005007341

[16] Basáñez MG, Remme JHF, Alley ES, Bain O, Shelley AJ, Medley GF, et al Density-dependent processes in the transmission of human onchocerciasis: relationship between the numbers of microfilariae ingested and successful larval development in the simuliid vector. Parasitology. 1995;110(4):409–427. 7753582 10.1017/s0031182000064751

[17] Basáñez MG, Townson H, Williams JR, Frontado H, Villamizar NJ, Anderson RM. Density-dependent processes in the transmission of human onchocerciasis: relationship between microfilarial intake and mortality of the simuliid vector. Parasitology. 1996;113 (4):331–355. 8873475 10.1017/s003118200006649x

[18] Dietz K. The population dynamics of onchocerciasis In: Anderson RM, editor. Population Dynamics of Infectious Diseases. London: Chapman and Hall; 1982 pp. 209–241.

[19] Basáñez MG, Boussinesq M. Population biology of human onchocerciasis. Philos Trans R Soc Lond B Biol Sci. 1999;354(1384):809–826. 10.1098/rstb.1999.0433 10365406 PMC1692549

[20] Duerr HP, Eichner M. Epidemiology and control of onchocerciasis: the threshold biting rate of savannah onchocerciasis in Africa. Int J Parasitol. 2010;40(6):641–650. 10.1016/j.ijpara.2009.10.016 19941867

[21] Filipe JAN, Boussinesq M, Renz A, Collins RC, Vivas-Martinez S, Grillet ME et al Human infection patterns and heterogeneous exposure in river blindness. Proc Natl Acad Sci U S A. 2005;102(42):15265–15270. 10.1073/pnas.0502659102 16217028 PMC1257694

[22] Basáñez MG, Walker M, Turner HC, Coffeng LE, de Vlas SJ, Stolk WA. River blindness: mathematical models for control and elimination. Adv Parasitol. 2016;94:247–341. 10.1016/bs.apar.2016.08.003 27756456

[23] Walker M, Stolk WA, Dixon MA, Bottomley C, Diawara L, Traoré MO, et al Modelling the elimination of river blindness using long-term epidemiological and programmatic data from Mali and Senegal. Epidemics. 2017;18:4–15. 10.1016/j.epidem.2017.02.005 28279455 PMC5340858

[24] Schofield SW, Sutcliffe JF. Humans vary in their ability to elicit biting responses from *Simulium venustum* (Diptera: Simuliidae). J Med Entomol. 1997; 34(1):64–67. 10.1093/jmedent/34.1.64 9086713

[25] Bockarie MJ, Davies JB. The transmission of onchocerciasis at a forest village in Sierra Leone. II. Man-fly contact, human activity and exposure to transmission. Ann Trop Med Parasitol. 1990; 84(6):599–605. 10.1080/00034983.1990.11812515 2076038

[26] Jacobi CA, Enyong P, Renz A. Individual exposure to *Simulium* bites and intensity of *Onchocerca volvulus* infection. Parasit Vect. 2010;3:53. 10.1186/1756-3305-3-53 PMC2910011 20565835

[27] Renz A, Fuglsang H, Anderson J. Studies on the dynamics of transmission of onchocerciasis in a Sudan-savanna area of North Cameroon IV. The different exposure to *Simulium* bites and transmission of boys and girls and men and women, and the resulting manifestations of onchocerciasis. Ann Trop Med Parasitol. 1987;81(3):253–262. 10.1080/00034983.1987.11812118 3662667

[28] Irvine MA, Reimer LJ, Njenga SM, Gunawardena S, Kelly-Hope L, Bockarie M, et al Modelling strategies to break transmission of lymphatic filariasis–aggregation, adherence and vector competence greatly alter elimination. Parasit Vectors. 2015;8:547 10.1186/s13071-015-1152-3 26489753 PMC4618540

[29] Irvine MA, Kazura JW, Hollingsworth TD, Reimer LJ. Understanding heterogeneities in mosquito-bite exposure and infection distributions for the elimination of lymphatic filariasis. Proc R Soc B. 2018; 285(1871). pii:20172253. 10.1098/rspb.2017.2253 PMC5805933 29386362

[30] Chandiwana SK, Woolhouse MEJ. Heterogeneities in water contact patterns and the epidemiology of *Schistosoma haematobium*. Parasitology. 1991;103(3):363–370. 1780173 10.1017/s0031182000059874

[31] Dye C, Hasibeder G. Population dynamics of mosquito-borne disease: effects of flies which bite some people more frequently than others. Trans R Soc Trop Med Hyg. 1986;80(1):69–77. 10.1016/0035-9203(86)90199-9 3727001

[32] Guelbéogo WM, Gonçalves BP, Grignard L, Bradley J, Serme SS, Hellewell J, et al Variation in natural exposure to *Anopheles* mosquitoes and its effects on malaria transmission. eLife. 2018;7:e32625 10.7554/eLife.32625 29357976 PMC5780040

[33] Winnen M, Plaisier AP, Alley ES, Nagelkerke NJD, van Oortmarssen G, Boatin BA et al Can ivermectin mass treatments eliminate onchocerciasis in Africa? Bull World Health Organ. 2002;(5):384–391. 12077614 PMC2567795

[34] Schulz-Key H, Karam M. Periodic reproduction of *Onchocerca volvulus*. Parasitol Today. 1986;2(10):284–286. 10.1016/0169-4758(86)90138-9 15462735

[35] Karam M, Schulz-Key H, Remme J. Population dynamics of *Onchocerca volvulus* after 7 to 8 years of vector control in West Africa. Acta Trop. 1987; 44(4):445–457. 2894134

[36] Duke BOL. The effects of drugs on *Onchocerca volvulus* I. Methods of assessment, population dynamics of the parasite and the effects of diethylcarbamazine. Bull World Health Organ. 1968; 39(2):137–146. 4881066 PMC2554552

[37] Plaisier AP, van Oortmarssen GJ, Remme J, Habbema JDF. The reproductive lifespan of *Onchocerca volvulus* in West African savanna. Acta Trop. 1991;48(4):271–284. 10.1016/0001-706x(91)90015-c 1674401

[38] Remme J, Ba O, Dadzie KY, Karam M. A force-of-infection model for onchocerciasis and its applications in the epidemiological evaluation of the Onchocerciasis Control Programme in the Volta River basin area. Bull World Health Organ. 1986;64(5):667–681. 3492300 PMC2490951

[39] Basáñez MG, Razali K, Renz A, Kelly D. Density-dependent host choice by disease vectors: epidemiological implications of the ideal free distribution. Trans R Soc Trop Med Hyg. 2007; 101(3):256–269. 10.1016/j.trstmh.2006.08.009 17112556

[40] Eichner M. *Onchocerca volvulus* (Nematoda, Filarioidea) und *Simulium damnosum*-Komplex (Diptera): Die Entwicklung intrathorakal injizierter Mikrofilarien in verschiedenen Überträgerspecies Kameruns. Diplomarbeit, Universität Tübingen, Fakultät für Biologie, Germany 1989 http://epimos.com/index.php?id=141&L=1.

[41] Coffeng LE, Stolk WA, Hoerauf A, Habbema D, Bakker R, Hopkins AD et al Elimination of African onchocerciasis: modelling the impact of increasing the frequency of ivermectin mass treatment. PLoS One. 2014;9(12):e115886 10.1371/journal.pone.0115886 25545677 PMC4278850

[42] Basáñez MG, Pion SDS, Boakes E, Filipe JAN, Churcher TS, Boussinesq M. Effect of single-dose ivermectin on *Onchocerca volvulus*: a systematic review and meta-analysis. Lancet Infect Dis. 2008; 8(5):310–322. 10.1016/S1473-3099(08)70099-9 18471776

[43] Plaisier AP, Alley ES, Boatin BA, van Oortmarssen GJ, Remme H, de Vlas SJ, et al Irreversible effects of ivermectin on adult parasites in onchocerciasis patients in the Onchocerciasis Control Programme in West Africa. J Infect Dis. 1995;172(1):204–210. 10.1093/infdis/172.1.204 7797912

[44] Renz A, Wenk P. Studies on the dynamics of transmission of onchocerciasis in a Sudan-savanna area of North Cameroon I. Prevailing *Simulium* vectors, their biting rates and age-composition at different distances from their breeding sites. Ann Trop Med Parasitol. 1987;81(3):215–228. 10.1080/00034983.1987.11812115 3662664

[45] Duke BOL, Anderson J, Fuglsang H. The *Onchocerca volvulus* transmission potentials and associated patterns of onchocerciasis at four Cameroon Sudan-savanna villages. Tropenmed Parasitol. 1975;26(2):143–154. 1172308

[46] Thylefors B, Philippon B, Prost A. Transmission potentials of *Onchocerca volvulus* and the associated intensity of onchocerciasis in a Sudan-savanna area. Tropenmed Parasitol 1978;29(3):346–354. 214908

[47] Wu J, Dhingra R, Gambhir M, Remais JV. Sensitivity analysis of infectious disease models: methods, advances and their application. J R Soc Interface. 2013;10(86):20121018 10.1098/rsif.2012.1018 23864497 PMC3730677

[48] Botto C, Basáñez MG, Escalona M, Villamizar NJ, Noya-Alarcón O, Cortez J, et al Evidence of suppression of onchocerciasis transmission in the Venezuelan Amazonian focus. Parasit Vectors. 2016;9:40 10.1186/s13071-016-1313-z 26813296 PMC4728794

[49] Vieira JC, Brackenboro L, Porter CH, Basáñez MG, Collins RC. Spatial and temporal variation in biting rates and parasite transmission potentials of onchocerciasis vectors in Ecuador. Trans R Soc Trop Med Hyg. 2005;99(3):178–195. 10.1016/j.trstmh.2004.03.012 15653120

[50] Vieira JC, Cooper PJ, Lovato R, Mancero T, Rivera J, Proaño R, et al Impact of long-term treatment of onchocerciasis with ivermectin in Ecuador: potential for elimination of infection. BMC Med. 2007;5:9 10.1186/1741-7015-5-9 17521449 PMC1890547

[51] Clopper C, Pearson ES. The use of confidence or fiducial limits illustrated in the case of the binomial. Biometrika. 1934;26(4):404–413.

[52] Efron B, Tibshirani RJ. An Introduction to the Bootstrap (Monographs on Statistics and Applied Probability) 1994, London: Chapman & Hall.

[53] O'Hanlon SJ, Slater HC, Cheke RA, Boatin BA, Coffeng LE, Pion SD, et al Model-based geostatistical mapping of the prevalence of *Onchocerca volvulus* in West Africa. PLoS Negl Trop Dis. 2016;10(1):e0004328 10.1371/journal.pntd.0004328 26771545 PMC4714852

[54] Dyson L, Stolk WA, Farrell SH, Hollingsworth TD. Measuring and modelling the effects of systematic non-adherence to mass drug administration. Epidemics. 2017;18:56–66. 10.1016/j.epidem.2017.02.002 28279457 PMC5340860

[55] Lamberton PHL, Cheke RA, Walker M, Winskill P, Crainey JL, Boakye DA, et al Onchocerciasis transmission in Ghana: the human blood index of sibling species of the *Simulium damnosum* complex. Parasit Vectors. 2016;9(1): 432 10.1186/s13071-016-1703-2 27494934 PMC4975878

[56] Quinnell RJ, Soremekun S, Bates PA, Rogers ME, Garcez LM, Courtenay O. Antibody response to sand fly saliva is a marker of transmission intensity but not disease progression in dogs naturally infected with *Leishmania infantum*. Parasit Vectors. 2018;11:7 10.1186/s13071-017-2587-5 29301571 PMC5755305

[57] World Health Organization/Department of Control of Neglected Tropical Diseases. Guidelines for stopping mass drug administration and verifying elimination of human onchocerciasis. Criteria and procedures. 2016; WHO/HTM/NTD/PCT/2016.1. http://apps.who.int/iris/bitstream/10665/204180/1/9789241510011_eng.pdf?ua=1. Accessed 28 May 2019. 26913317

[58] Golden A, Faulx D, Kalnoky M, Stevens E, Yokobe L, Peck R, et al Analysis of age-dependent trends in Ov16 IgG4 seroprevalence to onchocerciasis. Parasit Vectors. 2016; 9(1):338 10.1186/s13071-016-1623-1 27296630 PMC4907250

[59] Wang S, Spear RC. Exploring the contribution of host susceptibility to epidemiological patterns of *Schistosoma japonicum* infection using an individual-based model. Am J Trop Med Hyg. 2015;92(6):1245–1252. 10.4269/ajtmh.14-0691 25870427 PMC4458832

[60] Wang S, Spear RC. Exposure versus susceptibility as alternative bases for new approaches to surveillance for Schistosoma japonicum in low transmission environments. PLoS Negl Trop Dis. 2016;10(3):e0004425 10.1371/journal.pntd.0004425 26942912 PMC4778868

[61] Civitello DJ, Rohr JR. Disentangling the effects of exposure and susceptibility on transmission of the zoonotic parasite *Schistosoma mansoni*. J Anim Ecol. 2014;83:1379–1386. 10.1111/1365-2656.12222 24702134

[62] Turaga PS, Tierney TJ, Bennett KE, McCarthy MC, Simonek SC, Enyong PA, et al Immunity to onchocerciasis: cells from putatively immune individuals produce enhanced levels of interleukin-5, gamma interferon, and granulocyte-macrophage colony-stimulating factor in response to *Onchocerca volvulus* larval and male worm antigens. Infect Immun. 2000;68(4):1905–1911. 10.1128/iai.68.4.1905-1911.2000 10722581 PMC97365

[63] MacDonald AJ, Turaga PS, Harmon-Brown C, Tierney TJ, Bennett KE, McCarthy MC, et al Differential cytokine and antibody responses to adult and larval stages of *Onchocerca volvulus* consistent with the development of concomitant immunity. Infect Immun. 2002;70(6):2796–2804. 10.1128/IAI.70.6.2796-2804.2002 12010965 PMC127981

[64] Singleton DR, Stear MJ, Matthews L. A mechanistic model of developing immunity to *Teladorsagia circumcincta* infection in lambs. Parasitology. 2011;138(3):322–332. 10.1017/S0031182010001289 20946694

[65] Duerr HP, Dietz K, Schulz-Key H, Büttner DW, Eichner M. The relationships between the burden of adult parasites, host age and the microfilarial density in human onchocerciasis. Int J Parasitol. 2004;34(4):463–473. 10.1016/j.ijpara.2003.11.008 15013736

[66] Soboslay PT, Lüder CG, Hoffmann WH, Michaelis I, Helling G, Heuschkel C, et al 1994. Ivermectin-facilitated immunity in onchocerciasis; activation of parasite-specific Th1-type responses with subclinical *Onchocerca volvulus* infection. Clin Exp Immunol. 1994;96(2):238–244. 10.1111/j.1365-2249.1994.tb06548.x 8187332 PMC1534906

[67] Steel C, Lujan-Trangay A, Gonzalez-Peralta C, Zea-Flores G, Nutman TB. Immunologic responses to repeated ivermectin treatment in patients with onchocerciasis. J Infect Dis. 1991;164(3):581–287. 10.1093/infdis/164.3.581 1822959

[68] Allen JE, Adjei O, Bain O, Hoerauf A, Hoffmann WH, Makepeace BL, et al Of mice, cattle, and humans: the immunology and treatment of river blindness. PLoS Negl Trop Dis. 2008;2(4):e217 10.1371/journal.pntd.0000217 18446236 PMC2323618

[69] Njongmeta LM, Nfon CK, Gilbert J, Makepeace BL, Tanya VN, Trees AJ. Cattle protected from onchocerciasis by ivermectin are highly susceptible to infection after drug withdrawal. Int J Parasitol. 2004;34(9):1069–1074. 10.1016/j.ijpara.2004.04.011 15313133

[70] Glickman LT, Glickman NW, Moore GE, Lok JB, McCall JW, Lewis HB. Comparative effectiveness of sustained-release moxidectin (ProHeart 6) and ivermectin (Heartgard Plus) for the prevention of heartworm infection in dogs in the United States. Intern J Appl Res Vet Med. 2006;4(4):339–354.

[71] Plaisier AP, van Oortmarssen GJ, Habbema JDF, Remme J, Alley ES. ONCHOSIM: a model and computer simulation program for the transmission and control of onchocerciasis. Comput Methods Programs Biomed. 1990;31(1):43–56. 10.1016/0169-2607(90)90030-d 2311368

[72] Katabarwa MN, Eyamba A, Nwane P, Enyong P, Kamgno J, et al Fifteen years of annual mass treatment of onchocerciasis with ivermectin have not interrupted transmission in the west region of Cameroon. J Parasitol Res. 2013;2013:420928 10.1155/2013/420928 23691275 PMC3652197

[73] Kamga GR, Dissak-Delon FN, Nana-Djeunga HC, Biholong BD, Mbigha-Ghogomu S, Souopgui J, et al Still mesoendemic onchocerciasis in two Cameroonian community-directed treatment with ivermectin projects despite more than 15 years of mass treatment. Parasit Vectors. 2016;9(1):581 10.1186/s13071-016-1868-8 27842567 PMC5109673

[74] Katabarwa MN, Eyamba A, Nwane P, Enyong P, Yaya S, Baldiagaï J, et al Seventeen years of annual distribution of ivermectin has not interrupted onchocerciasis transmission in North Region, Cameroon. Am J Trop Med Hyg. 2001;85: 1041–1049. 10.4269/ajtmh.2011.11-0333 PMC3225149 22144441

[75] Katabarwa MN, Lakwo T, Habomugisha P, Agunyo S, Byamukama E, Oguttu D, et al Transmission of *Onchocerca volvulus* continues in Nyagak-Bondo focus of northwestern Uganda after 18 years of a single dose of annual treatment with ivermectin. Am J Trop Med Hyg. 2013;89:293–300. 10.4269/ajtmh.13-0037 23690555 PMC3741251

[76] Eisenbarth A, Achukwi MD, Renz A. Ongoing transmission of *Onchocerca volvulus* after 25 years of annual ivermectin mass treatments in the Vina du Nord River Valley, in North Cameroon. PLoS Negl Trop Dis. 2016;10(2):e0004392 10.1371/journal.pntd.0004392 26926855 PMC4771805

[77] Doyle SR, Bourguinat C, Nana-Djeunga HC, Kengne-Ouafo JA, Pion SDS, Bopda J, et al Genome-wide analysis of ivermectin response by *Onchocerca volvulus* reveals that genetic drift and soft selective sweeps contribute to loss of drug sensitivity. PLoS Negl Trop Dis. 2017;11(7):e0005816 10.1371/journal.pntd.0005816 28746337 PMC5546710

[78] Gardon J, Gardon-Wendel N, Demanga-Ngangue, Kamgno J, Chippaux JP, Boussinesq M. Serious reactions after mass treatment of onchocerciasis with ivermectin in an area endemic for *Loa loa* infection. Lancet. 1997;350(9070):18–22. 10.1016/S0140-6736(96)11094-1 9217715

[79] Kamgno J, Pion SDS, Chesnais CB, Bakalar MH, D'Ambrosio MV, Mackenzie CD, et al A test-and-not-treat strategy for onchocerciasis in *Loa loa*-endemic areas. N Engl J Med. 2017;377(21):2044–2052. 10.1056/NEJMoa1705026 29116890 PMC5629452

[80] Pion SDS, Clarke P, Filipe JAN, Kamgno J, Gardon J, Basáñez MG, et al Co-infection with *Onchocerca volvulus* and *Loa loa* microfilariae in central Cameroon: are these two species interacting? Parasitology. 2006;132(6): 843–854. 16469200 10.1017/S003118200600984X

[81] Walker M, Specht S, Churcher TS, Hoerauf A, Taylor MJ, Basáñez MG. Therapeutic efficacy and macrofilaricidal activity of doxycycline for the treatment of river blindness. Clin Infect Dis. 2015;60(8):1199–1207. 10.1093/cid/ciu1152 25537873 PMC4370165

[82] Opoku NO, Bakajika DK, Kanza EM, Howard H, Mambandu GL, Nyathirombo A, et al Single dose moxidectin versus ivermectin for *Onchocerca volvulus* infection in Ghana, Liberia, and the Democratic Republic of the Congo: a randomised, controlled, double-blind phase 3 trial. Lancet. 2018;392(10154):1207–1216. 10.1016/S0140-6736(17)32844-1 29361335 PMC6172290

[83] Routledge I, Walker M, Cheke RA, Bhatt S, Nkot PB, Matthews GA, et al Modelling the impact of larviciding on the population dynamics and biting rates of *Simulium damnosum* (s.l.): implications for vector control as a complementary strategy for onchocerciasis elimination in Africa. Parasit Vectors. 2018;11(1):316 10.1186/s13071-018-2864-y 29843770 PMC5972405

[84] Kuesel AC. Research for new drugs for elimination of onchocerciasis in Africa. Int J Parasitol Drugs Drug Resist. 2016;6(3):272–286. 10.1016/j.ijpddr.2016.04.002 27693536 PMC5196484

[85] Irvine MA, Hollingsworth TD. Kernel-density estimation and approximate Bayesian computation for flexible epidemiological model fitting in Python. Epidemics. 2018;25:80–88. 10.1016/j.epidem.2018.05.009 29884470 PMC6227249

