# Supplementary material for: Modelling exposure heterogeneity and density dependence in onchocerciasis using a novel individual-based transmission model, EPIONCHO-IBM: Implications for elimination and data needs
Source: PLoS Negl Trop Dis. 2019 Dec 5;13(12):e0007557. doi: 10.1371/journal.pntd.0007557 (PMC7006940; doi:10.1371/journal.pntd.0007557)
Supplement: S1 Text — A) Formal description of EPIONCHO-IBM. B) Uncertainty and sensitivity analyses using the Latin hypercube parameter sets. (DOCX) [file pntd.0007557.s001.docx]

**Modelling exposure heterogeneity and density dependence in onchocerciasis using a novel individual-based transmission model, EPIONCHO-IBM: implications for elimination and data needs**

Jonathan I. D. Hamley^1,2*†^, Philip Milton^1,2†^, Martin Walker^1,3‡^, Maria-Gloria Basáñez^1,2‡^

1. London Centre for Neglected Tropical Disease Research (LCNTDR), Department of Infectious Disease Epidemiology, School of Public Health, Faculty of Medicine (St Mary’s campus), Imperial College London, Norfolk Place, London W2 1PG, UK.

2. MRC Centre for Global Infectious Disease Analysis, Department of Infectious Disease Epidemiology, School of Public Health, Faculty of Medicine (St Mary’s campus), Imperial College London, Norfolk Place, London W2 1PG, UK.

3. London Centre for Neglected Tropical Disease Research (LCNTDR), Department of Pathobiology and Population Sciences, Royal Veterinary College, University of London, Hatfield AL9 7TA, UK.

^*^ Corresponding author: jonathan.hamley11@imperial.ac.uk

^†^Contributed equally, ^‡^Joint senior authors

**S1 Text: Detailed description of EPIONCHO-IBM and additional results**

**A. Formal description of EPIONCHO-IBM**

Here we describe the stochastic, individual-based analogue of the EPIONCHO transmission model parameterised for onchocerciasis transmission and control in the African savannah epidemiological and entomological context [1, 2]. Human stages of infection are modelled for each individual human host in the population using stochastic difference equations (adult *Onchocerca volvulus*) and deterministically using partial differential equations (microfilariae). Vector stages of infection (L1, L2 and L3 larvae) are modelled deterministically using a system of ordinary differential equations. Changes in the adult worm populations within an individual human are defined by stochastic transition events, where the number of events is drawn from a binomial distribution with the probability of a transition given by the product of the event rate multiplied by a time step $dt$. We introduce and define the notation for parasite state variables in Table A and throughout the subsequent description we use subscript $i$ to indicate quantities that are specific to an individual host, such as their exposure to blackfly bites. Subscripts $a$ and $s$ are context dependent, denoting the age and sex (respectively) of humans and parasites. Subscript $l$ (life stage) is used for rates in parasite life history, and is either $W$, for adult *O. volvulus* or $M$, for microfilariae (note $M$ is also context dependent and may refer to male humans or adult *O. volvulus*). The model was run in R, using the computer cluster at the Imperial Research Computing service [3].

**Table A. EPIONCHO-IBM state variable definitions.**

| State variable | Definition |
| --- | --- |
| $W_{s\left( i,a \right)}$ | Number of adult *Onchocerca volvulus* of sex $s$ ($s=F \mathrm{or}s=M)$ aged $a$ in human host $i$ |
| $W_{FN\left( i,a \right)}$ | Number of non-fertile adult female *O. volvulus* aged $a$ in human host $i$ |
| $W_{FF(i,a)}$ | Number of fertile adult female *O. volvulus* aged $a$ in human host $i$ |
| $M_{\left( i \right)}(a)$ | Number of *O. volvulus* microfilariae aged $a$ in human host $i$ |
| $L1$ | Mean number of L1 *O. volvulus* (sausage-stage) larvae per blackfly vector |
| $L2$ | Mean number of L2 *O. volvulus* (pre-infective) larvae per blackfly vector |
| $L3$ | Mean number of L3 *O. volvulus* (infective) larvae per blackfly vector |

**A.1. Human demography**

The human mortality rate is assumed to be constant with age, such that the probability of survival at age $a$, is given by

| $S_{H}\left( a \right)=e^{-a\mu_{H}}$ | (S1) |
| --- | --- |

It follows that the number of deaths at each time step is a random variable drawn from a binomial distribution, $binomal\left( N_{H}, \mu_{H}dt \right)$, where $N_{H}$ is the human population size. Exponential distributions are unbounded for positive values and thus the distribution is truncated to prevent excessively long lifespans; a maximum age, $a_{max}$, is implemented (80 years), at which all individuals die. We assume human births are exactly balanced by deaths, resulting in a constant, stable population size. Newborns are uninfected (i.e. there is no vertical transmission of parasites, although in utero transmission of microfilariae has been reported [4]). Individuals are assigned a sex $s=F$ or $s=M$ randomly, with an equal probability of assignment, i.e. $\psi_{F}^{'}=\psi_{M}^{'}=0.5$. (Note that we later also use subscript $s$ to denote the sex of adult parasites and hence the definition of $s$ is context dependent). Table B provides the definitions for human host demography.

**Table B. EPIONCHO-IBM parameter and variable definitions for human host demography.**

| Parameter or variable | Definition | Value and units | Reference |
| --- | --- | --- | --- |
| $N_{H}$ | Number of human hosts in population | 500 | This paper |
| $\mu_{H}$ | Mortality rate of human hosts | 0.02 year^-1^ | [1] |
| $a_{max}$ | Maximum age of human hosts | 80 years | [5] |
| $\psi_{S}^{'}$ | Probability that a human host is of sex $s$ | $\psi_{F}^{'}=\psi_{M}^{'}=0.5$ | [5] |

**A.2. Exposure to blackfly bites**

Individuals within the population are differentially exposed to blackfly bites depending on their age, sex, and an individual-specific exposure, $E_{(i)}$. This individual-specific exposure factor is assigned at birth and is drawn from a gamma distribution,

| $E_{(i)}\sim G(k_{E},\beta_{E})$ | (S2) |
| --- | --- |

where $k_{E}$ and $\beta_{E}$ are the shape and rate parameters, respectively. We assume always that $k_{E}$ = $\beta_{E}$, such that the mean exposure in the population is unity. This ensures that blackfly bites are distributed among hosts with an average exposure that is given by the annual biting rate (number of bites per person per year).

Exposure to blackfly bites is also a function of host age and sex. Following [5],

| $\Omega_{s}(a_{(i)})=E_{s}\cdot\gamma_{s}\cdot E_{0} \mathrm{for}a_{\left( i \right)}<q$  $\Omega_{s}(a_{(i)})=E_{s}\cdot\gamma_{s}\cdot{exp}^{\left[ -\alpha_{s}\left( a_{\left( i \right)}-q \right) \right]} \mathrm{for} a_{\left( i \right)}\geq q$ | (S3) |
| --- | --- |

where $E_{s}$ is the sex-specific exposure to vector bites (calculated from the relative exposure of males versus females $Q={E_{M}}/{E_{F}})$; $E_{0}$is the fraction of exposure at age 0 relative to the age from which exposure changes continuously with age (when $a_{\left( i \right)}\geq q$), and$\alpha_{s}$ is the sex-specific change in contact rate between vectors and hosts with increasing host age*.* This is multiplied by the normalisation factor $\gamma_{s}$ to ensure that the mean exposure in the population is unity. An individual’s relative age- and sex-specific exposure is combined with their individual-specific biting factor to produce their total exposure to blackfly bites,

| $\Omega_{T}(a_{\left( i \right)})=E_{(i)}\Omega_{s}(a_{\left( i \right)})$ | (S4) |
| --- | --- |

Table C summarises the parameters and variable definitions for human exposure to vector bites.

**Table C. EPIONCHO-IBM parameter and variable definitions for exposure to blackfly bites.**

| Parameter or variable | Definition | Value and units | Reference |
| --- | --- | --- | --- |
| $k_{E},\beta_{E}$ | Shape and rate parameters of the gamma distribution describing individual human host exposure to blackfly bites | Vary | This paper |
| $Q={E_{M}}/{E_{F}}$ | Relative male to female exposure to blackfly bites | 1.20 | [5] |
| $\alpha_{F}$ | Age-specific change in contact rate with vectors for females | -0.023  year^-1^ | [5] |
| $\alpha_{M}$ | Age specific change in contact rate with vectors for males | 0.007  year^-1^ | [5] |
| $q$ | Period (age) preceding initial increase in exposure to vector bites during childhood | 0 years | [5] |

The values of these parameters pertain to the savannah epidemiological setting of northern Cameroon, where the vectors are *Simulium damnosum* s.s.*/S. sirbanum*.

**A.3. Human host infection**

Human host infection dynamics are modelled by stochastic and deterministic simulation. The rate at which infective L3 larvae in vectors are acquired by the host is governed by the annual transmission potential (ATP), which is calculated as the number of larvae received per person per year. The ATP at time $t$ is the product of three factors, namely, the per blackfly biting rate on humans, $\beta=h/g$ (where $h$ is the human blood index, times the fraction of bloodmeals taken on humans, and $g$ is the duration of the gonotrophic cycle, expressed in years, taken as the period between two consecutive bloodmeals and assuming gonotrophic concordance, i.e. one bloodmeal leads to the production of one batch of eggs), the ratio of vectors to human hosts, $V/H$, and the temporally dynamic mean number of infective L3 larvae per blackfly vector, i.e. $ATP\left( t \right)=\beta\left( V/H \right)L3\left( t \right)$. The quantity $V/H$ is inferred from the annual biting rate $ABR= \beta V/H$, which is the key input for adjusting the endemicity level of EPIONCHO-IBM simulations. Establishment of L3 larvae in human hosts is negatively density (transmission intensity) dependent, with the per capita rate of worm establishment decreasing with increasing ATP [1, 2, 5, 6], following [7]. This density dependence is controlled by the function $\Pi_{H(i)}\left[ ATP\left( t-\tau_{H} \right),\Omega_{T}{(a}_{(i)}-\tau_{H}) \right]$(Table D) representing the (decreasing) proportion of L3 larvae developing into adult worms within the human host as a function of (increasing) ATP. We assume a delay ($\tau_{H}$) between L3 larvae entering the host and establishing as adult worms to account for the duration of development into L4 stages and L5 (juvenile adults). Additionally, we model the severity of density dependence as an individual-level property.

Thus, the individual rate of acquisition of male and female adult worms (where sex is denoted by subscript $s=F$ or $s=M$) is modelled as a discrete-time stochastic process,

| $W_{s(i,1)}\left( t \right)\to W_{s(i,1)}\left( t+dt \right) + 1-$ | $with probability:\psi_{s}{\Omega_{T}{(a}_{(i)}-\tau_{H})ATP\left( t-\tau_{H} \right){\Pi_{H}}_{(i)}\left[ ATP\left( t-\tau_{H} \right),\Omega_{T}{(a}_{(i)}-\tau_{H}) \right]}dt$ | (S5) |
| --- | --- | --- |

where $W_{s(i,1)}$ is the number adult worms of sex $s$ in the first age group, and $\psi_{s}$ is the (equal) probability that an incoming parasite is male or female, such that $\psi_{M}= \psi_{F}=0.5$ [8]. Table D describes the parameters and variables determining human host infection.

**Table D. EPIONCHO-IBM parameter and variable definitions for human host infection.**

| Parameter or variable | Definition | Value and units | Reference |
| --- | --- | --- | --- |
| $\beta=h/g$ | Per blackfly biting rate on humans, calculated as the product of the proportion of blackfly bites taken on humans (the human blood index, *h*) and the reciprocal of the duration of the gonotrophic cycle, *g* | $h=$ 0.63^§^  $g=1/104$  years | [1, 9] |
| $ABR= \beta V/H$ | Annual biting rate of blackflies on humans; the key variable for simulating different endemicity levels | Varies;  bites/person/year | This paper |
| $ATP\left( t \right)=ABR\times L3\left( t \right)$ | Annual transmission potential of blackflies to humans | Defined by $ABR$ and $L3\left( t \right)$ | [1] |
| $\Pi_{H\left( i \right)}\left[ ATP\left( t-\tau_{H} \right),\Omega_{T}{(a}_{\left( i \right)}-\tau_{H}) \right]=\left[ \frac{{\delta_{H0}+\delta}_{H\infty}c_{H}ATP\left( t-\tau_{H} \right)\Omega_{T}{(a}_{(i)}-\tau_{H})}{1+c_{H}ATP\left( t-\tau_{H} \right)\Omega_{T}{(a}_{(i)}-\tau_{H})} \right]$ | Density-dependent constraint on the proportion of infective L3 larvae successfully establishing as adult worms | defined by $\delta_{H0}$, $\delta_{H\infty}$, $c_{H}$, $ATP\left( t-\tau_{H} \right)$ and $\Omega_{T}(a_{(i)}-\tau_{H})$ | [1,5,6] |
| $\delta_{H0}$ | Proportion of L3 larvae developing to the adult stage within the human host, per bite, when $ATP\left( t \right)\to0$ | Dimensionless, varies | Re-estimated in this paper |
| $\delta_{H\infty}$ | Proportion of L3 larvae developing to the adult stage within the human host, per bite, when $ATP\left( t \right)\to\infty$ | Dimensionless, varies | Re-estimated in this paper |
| $c_{H}$ | Severity of transmission intensity-dependent parasite establishment within humans | varies | Re-estimated in this paper |
| $\tau_{H}$ | Time delay between L3 entering the host and establishing as adult worms | 0.8 years | [10] |

^§^The value of the proportion of vector bloodmeals taken on humans corresponds to *Simulium damnosum* s.s./ *S. sirbanum* as reported in [9].

**A.4. Parasite demography**

***A.4.1 Parasite mortality.*** The mortality rates of both adult worms and microfilariae are assumed to increase as a function of parasite age, according to a Weibull distribution of survival times,

| $\mu_{l}\left( a \right)= {y_{l}}^{d_{l}}d_{l}a^{d_{l}-1}$ |  | (S6) |
| --- | --- | --- |

| $S_{l}\left( a \right)=e^{{-y_{l}a}^{d_{l}}}$ |  | (S7) |
| --- | --- | --- |

where $l$ denotes the parasite life stage $W$ (adult worms) or $M$ (microfilariae). Parameters $y_{l}$ and $d_{l}$ were estimated by fitting Eq (S7) to the data in [11] (for adult worms, Fig A (a)) and in [12] (for microfilariae, Fig A (b)). The life expectancy of each life stage is given by $\bar{L_{l}}= \int_{0}^{\infty} S_{l}\left( a \right)da$.

**Fig A. Survivorship of adult *Onchocerca volvulus* (a) and microfilariae (b).** Adult and microfilarial survival were estimated by fitting Eq (S7) (black lines) to the data (red points) in [11] (adults) and [12] (microfilariae). Eq (S7) was fitted by minimising the sum of squared residuals. The adult worm data are percentages of nodules with live worms in OCP village clusters after vector control. The microfilarial data pertain to the proportion of microfilariae surviving at various times after treatment with potassium melarsonyl (a known macrofilaricidal drug with negligible microfilaricidal activity), measured in two patients infected with *O. volvulus.*

Assuming a maximum number of discrete age classes $c_{max}$, and a maximum life span for each life stage $L_{l}$, we adjusted each age class to the appropriate duration, $q_{l}$, where $l$ is the parasite life stage. Worm mortality and movement between age classes is modelled, like other transitions, as a discrete-time stochastic process. Worms leave an age class either due to death or progression to the next age class following,

| $W_{s\left( i,a \right)}\left( t \right)\to W_{s\left( i,a \right)}\left( t+dt \right)-1$  $W_{s\left( i,a+q_{W} \right)}\left( t \right)\to W_{s\left( i,a+q_{W} \right)}\left( t+dt \right)+1$ | $With probabilities:$  ${\mu_{W}\left( a \right)W}_{s\left( i,a \right)}dt$  $\frac{W_{s\left( i,a \right)}dt}{q_{W}}$  for $a \leq L_{W}$  $with probability: \frac{W_{s\left( i,a \right)}dt}{q_{W}}$  for $0<a<L_{W}$ | (S8) |
| --- | --- | --- |

***A.4.2 Parasite fecundity and fertility.*** Newly established adult female *O. volvulus*, are initially non-fertile,$W_{FN\left( i, a \right)}$, and progress to become fertile, $W_{FF\left( i, a \right)}$ [13], at a per capita rate $\omega$. Female worms only produce microfilariae when in the fertile state and in the presence of at least one co-infecting male worm (assuming complete polygamy of male worms such that one male can mate with all females within the same human host) [8,14]. The stochastic process governing the rate of progression to fertility is defined as,

| $W_{FN(i,a)}\left( t \right)\to W_{FN(i,a)}\left( t+dt \right)- 1,$  $W_{FF\left( i,a \right)}\left( t \right)\to W_{FF\left( i,a \right)}\left( t+dt \right)+ 1$ | $with probability:\omega W_{FN\left( i,a \right)}dt$ | (S9) |
| --- | --- | --- |

Because female worms need to be re-inseminated at each reproductive cycle [8,15], we further assume that fertile female worms lose their fertility and return to the non-fertile state at the per capita rate, $\lambda_{0}$, such that,

| $W_{FN(i,a)}\left( t \right)\to W_{FN(i,a)}\left( t+dt \right)+ 1,$  $W_{FF\left( i,a \right)}\left( t \right)\to W_{FF\left( i,a \right)}\left( t+dt \right)-1$ | $with probability:{\lambda_{0}W}_{FF(i,a)}dt$ | (S10) |
| --- | --- | --- |

The contribution of each age class of adult female worms to the number of microfilariae in individual $i$ depends on the fecundity rate, $m\left( a \right)$, and on the presence of at least one male worm,

| $m\left( a \right)=\frac{\varepsilon^{*}F}{F+G^{-a}-1}$  $m\left( a \right)=0$ | for $\sum_{a=0}^{a=L_{W}} W_{M\left( i,a \right)}\left( t \right)> 0$  for $\sum_{a=0}^{a=L_{W}} W_{M\left( i,a \right)}\left( t \right)= 0$ | (S11) |
| --- | --- | --- |

where $\varepsilon^{*}$ is the fecundity rate at age 0, and $F$ and $G$ determine the decline in fecundity with worm age. Eq (S11) was parameterised to mimic, with a nonlinear function, the modelled age-dependent fecundity assumed by Plaisier et al. [16]. They assumed that female worms have maximum fecundity during the first 5 years of age followed by a linear reduction in fecundity which becomes zero for worms older than 20 years. Fig B illustrates the shape of the age-dependent fecundity function incorporated into EPIONCHO-IBM.

**Fig B. Age-dependent fecundity rate of (fertile) adult female *Onchocerca volvulus*.** The solid lines depict the behaviour of Eq (S11), motivated by the assumptions made in [16]. The red solid line corresponds to the function used in EPIONCHO-IBM, with values for F and G equal to, respectively, 70 and 0.72. The black solid lines illustrate other behaviours obtained by varying G (0.57 (bottom line), 0.62, 0.67, 0.77, 0.82 (top line)).

To test the combined effect of the assumptions of age-dependent adult worm mortality, microfilarial mortality and female worm fecundity, the community microfilarial load (CMFL, the geometric mean no. of microfilariae per skin snip in those aged ≥ 20 years [17]) predicted by EPIONCHO-IBM was fitted (altering only the ABR to reflect transmission conditions before intervention) to the data presented in [16] on temporal trends following vector control in villages of the Onchocerciasis Control Programme in West Africa (OCP) (Fig C).

**Fig C. EPIONCHO-IBM and observed trends in community microfilarial load (CMFL [17]) following the introduction of vector control by the Onchocerciasis Control Programme in West Africa.** The model was validated using data collected since the introduction of vector control in Burkina Faso and presented in [16], by altering the annual biting rate (ABR) before intervention and using $\delta_{H0}$ = 0.186, $\delta_{H\infty}$ = 0.003, $c_{H}$ = 0.005 and $k_{E}$ = 0.3 (parameters estimated in this paper). The solid black line corresponds to the model prediction and the solid red circles to the data from the communities of (a) Tiercoura, (b) Loaba, (c) Folonzo and (d) Sarba Baforo.

The change in the density of microfilariae per mg of skin is calculated deterministically by the partial differential equation,

| $\frac{\partial M_{(i)}}{dt}+ \frac{\partial M_{(i)}}{da}= {-\mu_{M}\left( a \right)M}_{\left( i \right)}(a,t)$ | (S12) |
| --- | --- |

where $M_{\left( i \right)}(0, t)$ =$\sum_{a=0}^{a=L_{W}} W_{FF\left( i, a \right)}\left( t \right)m\left( a \right)$, the sum of the reproductive output of all fertile adult female worms within a host. Note that computationally, we discretise microfilarial age classes in the same way as adult worms (depending on $c_{max}$, $L_{M}$, $q_{M}$), but calculate changes in the microfilarial density by age and time using the fourth-order Runge-Kutta method. All parameters and variables in EPIONCHO-IBM that describe parasite demography are summarised in Table E.

**Table E. EPIONCHO-IBM parameter and variable definitions for parasite demography.**

| Parameter  or variable | Definition | Value and units | Reference |
| --- | --- | --- | --- |
| $y_{W}$ | Parameter relating mortality rate to age in adult worms, Eq (S6), (S7) | 0.1 | This paper |
| $d_{W}$ | Parameter relating mortality rate to age in adult worms, Eq (S6), (S7) | 6.01 | This paper |
| $y_{M}$ | Parameter relating mortality rate to age in microfilariae, Eq (S6), (S7) | 1.09 | This paper |
| $d_{M}$ | Parameter relating mortality rate to age in microfilariae, Eq (S6), (S7) | 1.43 | This paper |
| $L_{W}$ | Maximum longevity of adult worms | 20 years | [16] |
| $L_{M}$ | Maximum longevity of microfilariae | 2.5 years | [12] |
| $c_{max}$ | Number of discrete age classes in adult worms and microfilariae | 21 | This paper |
| $q_{M}$ | Duration of each age class for microfilariae | 0.125 years | This paper |
| $q_{W}$ | Duration of each age class for adult worms, Eq (S8) | 1 year | This paper |
| $\varepsilon^{*}$ | Per capita rate of production of microfilariae per mg of skin per (fertile) adult female *Onchocerca volvulus* at age zero, Eq (S11) | 1.15 year^-1^ | [2] |
| $\omega$ | Per capita rate of progression from non-fertile to fertile adult female *O. volvulus,* Eq (S9) | 0.59 year^-1^ | [2,13] |
| $\lambda_{0}$ | Per capita rate of reversion from fertile to non-fertile adult female *O. volvulus,* Eq (S10) | 0.33 year^-1^ | [2,13] |
| $F$ | Parameter relating parasite fecundity to age, Eq (S11) | 70 | This paper |
| $G$ | Parameter relating parasite fecundity to age, Eq (S11) | 0.72 | This paper |

**A.5. Larval and vector population dynamics**

The vector transmission cycle is modelled deterministically. An individual’s contribution to the larval burden in the vector population (subscript *V*) is calculated as a function of their microfilarial load. This contribution depends on the biting rate per fly on humans, and the individual’s specific relative exposure to blackfly bites, $\Omega_{T}(a_{\left( i \right)})$*.* Much like the establishment of L3 larvae in the human host, the establishment of microfilariae in the simuliid vector is determined by a constraining density-dependent function. This density dependence is defined by $\Pi_{V(i)}(t)$ (Table F), such that the proportion of microfilariae developing into infective L3 larvae within the blackfly vector declines with increasing number of microfilariae ingested [1, 2]. Additionally, this proportion is affected by the probability that a blackfly survives the extrinsic incubation period (EIP), the time it takes for ingested microfilariae to develop into L3 larvae, which represents a substantial proportion of the blackfly’s lifespan. The EIP is incorporated by modelling explicitly the L1, L2 and L3 stages of development within the blackfly vector [18, 19] whilst also accounting for a delay ($\tau_{V}$) before L1 can start transitioning to the L2 stage, as suggested by experimental blackfly infection data [20]. The within-blackfly dynamics of L1, L2 and L3 stages are defined by,

| $\frac{d{L1}_{(i)}\left( t \right)}{dt}=\beta\Pi_{V\left( i \right)}(t)\Omega_{T}(a_{\left( i \right)})M_{\left( i \right)}\left( t \right)- {L1}_{\left( i \right)}\left( t \right){(\mu}_{V}+ \alpha_{V}\cdot M_{\left( i \right)}\left( t \right)\cdot\Omega_{T}(a_{\left( i \right)}))- {L1}_{\left( i \right)}\left( t-\tau_{V} \right) \nu_{1} e^{{-\tau_{V}(\mu}_{V}+ \alpha_{V}\cdot M_{\left( i \right)}\left( t-\tau_{V} \right)\cdot\Omega_{T}(a_{\left( i \right)}-\tau_{V}))}$ | (S13) |
| --- | --- |
| $\frac{d{L2}_{(i)}\left( t \right)}{dt}={L1}_{\left( i \right)}\left( t-\tau_{V} \right) \nu_{1} e^{{-\tau_{V}(\mu}_{V}+ \alpha_{V}\cdot M_{\left( i \right)}\left( t-\tau_{V} \right)\cdot\Omega_{T}(a_{\left( i \right)}-\tau_{V}))}- {L2}_{\left( i \right)}\left( t \right)\left( {\nu_{2}+ \mu}_{V} \right)$ | (S14) |
| $\frac{d{L3}_{(i)}\left( t \right)}{dt}={\nu_{2}L2}_{\left( i \right)}\left( t \right)- {L3}_{\left( i \right)}\left( t \right)\left( \mu_{V}+ \mu_{L3}+\left( \frac{a_{H}}{g} \right) \right)$ | (S15) |

Here, L1 larvae develop into L2 larvae at per capita rate $\nu_{1}$ (estimated from *Simulum damnosum* s.l. data from Cameroon in [20]) if they survive for at least time $\tau_{V}$, or are lost when a blackfly vector dies due to the natural and parasite induced mortality rates. The life expectancy of vectors is dependent on the number of microfilariae ingested per bite, the third constraining density-dependent process modelled in the parasite life-cycle. This parasite-induced excess mortality of blackflies is included as the product of a per microfilaria excess mortality term,$\alpha_{V}$, multiplied by the number of microfilariae ingested $M_{(i)}$ [1,2,21]. Infective L3 larvae develop from L2 (pre-infective larvae) at per capita rate $\nu_{2}$ [20] and are lost from vectors as a result of three possible processes, namely, at per capita mortality rate $\mu_{L3}$, when a blackfly dies at per capita rate $\mu_{V},$or upon inoculation into a vertebrate host (including humans) when a blackfly bites. The latter rate is given by the product of the proportion of L3 shed per bite (on any blood host), $a_{H}$, and the per capita rate of biting$1/g$, where $g$ is the length of the gonotrophic cycle [1,2]. The mean number of L3 per blackfly is denoted $L3= \sum_{i} {L3}_{i}/N$. This provides the dynamic input to the ATP expression which defines the human hosts’ annual rate of acquisition of new parasites in Eq (S5). Table F provides the description of parameters and variables for the dynamics for the vector stages of *O. volvulus* larvae and the adult female blackfly population. (Note that the stages of the vector population are not explicitly modelled in this paper but see [22, 23] for the description of a deterministic model of *S. damnosum* s.l. dynamics.)

**Table F. EPIONCHO-IBM parameter and variable definitions for larval stages within the vector and adult female blackfly population dynamics.**

| Parameter or variable | Definition | Value and units | Reference |
| --- | --- | --- | --- |
| $\Pi_{V(i)}\left( t \right)=\frac{\delta_{V0}}{\left[ 1+c_{V}M_{(i)}\left( t \right)\Omega_{T}(a_{\left( i \right)}) \right]}$ | Proportion of microfilariae (mf) per mg of skin in human host $i$ developing into infective L3 larvae within the blackfly vector per bite | Defined by $\delta_{V0}$, $c_{V}$, $M_{(i)}\left( t \right)$ and $\Omega_{T}(a_{\left( i \right)})$ | [1, 2] |
| $\delta_{V0}$ | Proportion of mf per mg developing to the infective L3 stage per bite when $M_{(i)}\left( t \right)\to0$ | Dimensionless, 0.0207 | [2] |
| $c_{V}$ | Severity of constraining density-dependent larval development per dermal microfilaria | 0.00878 | [1] |
| $\nu_{1}$ | Per capita development rate from L1 to L2 larvae | 201.6 year^-1^ | [20] |
| $\nu_{2}$ | Per capita development rate from L2 to L3 larvae | 207.7 year^-1^ | [20] |
| $\mu_{V}$ | Per capita mortality rate of blackfly vectors | 26 year^-1^ | [1, 2] |
| $\alpha_{V}$ | Per capita microfilaria-induced mortality of blackfly vectors | 0.39 year^-1^ | [1] |
| $\tau_{V}$ | Delay before L1 larvae can start transitioning to L2 stages | 0.011 years  (4 days) | [20] |

**A.6. Treatment with ivermectin**

The pharmacodynamics of skin microfilarial load and the proportion of adult female worms producing live microfilariae following ivermectin treatment (with the standard dose of 150 μg/kg dose) are modelled according to the parameterisation presented in [13]. The microfilaricidal effect is modelled as a per capita excess mortality rate $\mu_{M(i)}^{'}\left( \tau_{h\left( i \right)} \right)$ that depends on the time elapsed since treatment,$\tau_{h\left( i \right)}$. Note that since an individual will not necessarily take treatment at each round, there will be variation in $\tau_{h\left( i \right)}$ in the human population. Hence, the dynamics of the microfilarial population following treatment with ivermectin is modelled by generalising Eq (S12) to

| $\frac{\partial M_{(i)}}{dt}+ \frac{\partial M_{(i)}}{da}= {-\left( \mu_{M}\left( a \right)+\mu_{M(i)}^{'}(\tau_{h\left( i \right)}) \right) M}_{\left( i \right)}(a,t)$ | (S16) |
| --- | --- |

where $\mu_{M(i)}^{'}\left( \tau_{h(i)} \right)={(\tau_{h(i)}+u)}^{-\kappa}$. This function permits a very large but finite microfilaricidal effect at the time of treatment, defined by $u$, followed by a decline, with the shape of this decline governed by$\kappa$. The temporary sterilisation of adult female worms (the so-called embryostatic effect) following treatment with ivermectin is modelled by including a treatment-induced excess per capita rate $\lambda_{(i)}^{'}\left( \tau_{h\left( i \right)} \right)$ at which fertile females become non-fertile. Hence, female worms move between the fertile and non-fertile compartments at an additional rate,

| $W_{FN\left( i,a \right)}\left( t \right)\to W_{FN\left( i,a \right)}\left( t+dt \right)+ 1,$  ${W_{FF}}_{\left( i,a \right)}\left( t \right)\to{W_{FF}}_{\left( i,a \right)}\left( t+dt \right)- 1$ | $with probability:{\lambda_{(i)}^{'}\left( \tau_{h\left( i \right)} \right)W_{FF}}_{\left( i,a \right)}dt$ | (S17) |
| --- | --- | --- |

Here, $\lambda_{(i)}^{'}\left( \tau_{h\left( i \right)} \right)=\lambda^{max}e^{\left( -\varphi{\tau_{h}}_{\left( i \right)} \right)}$, where $\lambda^{max}$ is the maximum rate of treatment-induced sterility and $\varphi$ is the rate of decay of this effect with time after treatment. We further assume that for each treatment from the second round onwards, a proportion of adult worms (in both the fertile and non-fertile compartments), $\lambda_{p}^{'}$, are made permanently infertile [24]. Table G provides definitions and values of the parameters and variables that determine the parasite dynamics following ivermectin administration.

**Table G. EPIONCHO-IBM parameter and variable definitions for ivermectin treatment*.***

| Parameter or variable | Definition | Value and units | Reference |
| --- | --- | --- | --- |
| $\mu_{M(i)}^{'}\left( \tau_{h(i)} \right)={(\tau_{h(i)}+u)}^{-\kappa}$ | Ivermectin-induced per capita rate of excess mortality of microfilariae at time $\tau_{h(i)}$ since treatment, Eq (S16) | Defined by $u$ and $\kappa$ | [13] |
| $u$ | Constant to allow for very large yet finite microfilaricidal effect upon treatment with ivermectin | 9.6×10^-3^ | [13] |
| $\kappa$ | Shape parameter for excess microfilarial mortality following treatment with ivermectin | 1.25 | [13] |
| $\lambda_{(i)}^{'}\left( \tau_{h(i)} \right)=\lambda^{max}e^{\left( -\varphi\tau_{h(i)} \right)}$ | Ivermectin-induced per capita rate of reversion from fertile to non-fertile adult female *O. volvulus* at time $\tau_{h(i)}$ since the last treatment, Eq (S17) | Defined by $\lambda^{max}$ and $\varphi$ | [13] |
| $\lambda^{max}$ | Maximum rate of ivermectin-induced female worm sterility | 32.4 year^-1^ | [13] |
| $\varphi$ | Rate of decay of ivermectin-induced female worm sterilisation | 19.6 year^-1^ | [13] |
| $\lambda_{p}^{'}$ | Proportion of adult female worms made permanently infertile at each ivermectin treatment round | 0.345 | [24] |

**A.7. Microfilarial intensity and prevalence, and skin snip sensitivity**

Microfilariae are assumed to be aggregated within human skin [25], such that that the *observed* number of microfilariae $M_{(i)}^{*}\left( t \right)$ in a single skin snip (the method used to detect the presence and quantify the intensity of microfilaridermia [26]) of weight $w$ is a random variable following a negative binomial distribution (NBD) with (modelled) mean $M_{(i)}\left( t \right)w$ and overdispersion (aggregation) parameter $k_{M(i)}$,

| $M_{(i)}^{*}\left( t \right)\sim\text{NBD}\left( M_{(i)}\left( t \right)w,k_{M(i)} \right)$ |  | (S18) |
| --- | --- | --- |

The degree of aggregation of skin microfilariae is assumed to *decrease* with increasing numbers of adult female worms harboured by the host and is parameterised as,

| $k_{M\left( i \right)}=0.0478{\times W}_{F\left( i \right)}+0.313$ |  | (S19) |
| --- | --- | --- |

using estimates of the overdispersion parameter and mean number of adult female worms per host reported in [27]. We also validated this model using independent data on microfilarial counts from 40 repeated skin snips taken from 15 individuals over 24 hours as reported in [28] (Fig D).

**Fig D. Validation of the model describing aggregation (overdispersion) of microfilariae in the human host skin.** Data points represent the variance and mean of 40 skin snips taken over 24 hours from 15 patients as reported in [28]. The solid black line is the predicted variance vs. mean relationship from the model developed in [27] and used in EPIONCHO-IBM with aggregation parameter linearly related to the number of female worms per host according to Eq (S19).

Typically, $n$ skin snips (in this paper $n=2$) are taken from each host to estimate the microfilarial density per milligram of skin, $\bar{M}_{(i)}^{*}\left( t \right)$. Hence, for $k=1\ldots n$ skin snips of weight $w$,

| $\bar{M}_{(i)}^{*}\left( t \right)= \frac{1}{nw}\sum_{k=1}^{k=n} M_{(i,k)}^{*}\left( t \right)$ |  | (S20) |
| --- | --- | --- |

and the mean number of mf per mg of skin per human host is,

| $\bar{M}^{*}\left( t \right)= \frac{1}{N_{H}}\sum_{i=1}^{i=N_{H}} \bar{M}_{(i)}^{*}\left( t \right)$ |  | (S21) |
| --- | --- | --- |

Prevalence is calculated in the same manner, converting $\bar{M}_{(i)}^{*}$to a binary variable such that,

| $\bar{P}_{\left( i \right)}^{*}\left( t \right)=1$ | $\mathrm{for}\bar{M}_{(i)}^{*}>0 and 0 otherwise$ | (S22) |
| --- | --- | --- |

and

| $\bar{P}^{*}\left( t \right)= \frac{1}{N_{H}}\sum_{i=1}^{i=N_{H}} \bar{P}_{(i)}^{*}\left( t \right)$ |  | (S23) |
| --- | --- | --- |

**Table H. EPIONCHO-IBM parameter and variable definitions for skin microfilarial intensity (density) and prevalence.**

| Parameter or variable | Definition | Value and units | Reference |
| --- | --- | --- | --- |
| $M_{(i,k)}^{*}\left( t \right)$ | The observed number of microfilariae in a single skin snip $k$ from human host $i$ | Model output | This paper |
| $w$ | The average weight of skin for one skin snip (taken with a Holth-type corneoscleral punch [27]) | 2 mg | [2, 7] |
| $\bar{M}_{(i)}^{*}\left( t \right)$ | The mean number of microfilariae per mg skin from $n$ skin snips of weight $w$ in human host $i$ | Model output | This paper |
| $\bar{P}_{(i)}^{*}\left( t \right)$ | A binary variable indicating positivity for microfilariae in human host $i$ | Model output | This paper |
| $n$ | The number of skin snips taken per individual human host | $2$ | This paper |
| $\bar{M}^{*}\left( t \right)$ | The mean number of microfilariae per mg of skin per human host | Model output | This paper |
| $k_{M\left( i \right)}=k_{M0}+k_{M1}W_{F\left( i \right)}$ | The degree of microfilarial aggregation within the skin of human host $i$ | defined by $k_{M0}$ and $k_{M1}$ | This paper |
| $k_{M0}$ | The degree of microfilarial aggregation in the skin as $W_{F\left( i \right)}\left( t \right)\to0$ | 0.313 | This paper |
| $k_{M1}$ | The change in microfilarial aggregation with increasing$W_{F\left( i \right)}\left( t \right)$ | 0.048 per adult female worm | This paper |

**B. Uncertainty and sensitivity analyses using the Latin hypercube parameter sets**

In order to assess the ability of the parameter ranges used for Latin Hypercube sampling (LHS) to capture the parasitological data, we plotted the model predicted microfilarial prevalence and intensity for each parameter set (for exposure heterogeneity parameter $k_{E}$ = 0.3) against the data used for fitting (Fig E (a) and (b)) (the fitting datasets are those from savannah settings in Cameroon [29, 31], and Burkina Faso/Côte d’Ivoire [30]). Most of the data are within the whiskers (minimum and maximum values), indicating that the LHS captured the data well. Partial rank correlation coefficients (PRCC) can be used to understand the influence of individual parameters, when more than one is changed simultaneously, as is the case with Latin hypercube sampling. PRCC values near 1 or -1, indicate influential parameters, whereas values near 0 indicate less influential parameters. In short, the PRCC removes the correlation between parameters, excluding the one under consideration; a full overview of this method can be found in [32]. Fig F shows the PRCCs for three annual biting rates, indicating the influence of parameters on the (per-intervention) microfilariae per mg of skin. The influence of $\delta_{H0}$ decreases as biting rate increases, whilst $\delta_{H\infty}$ is more influential over model predictions at higher biting rates. Because low annual biting rates are associated with low annual transmission potentials, they result in a high proportion of parasites establishing in the human host, i.e. towards the left of the relationship between $\Pi_{H\left( i \right)}\left[ ATP\left( t-\tau_{H} \right),\Omega_{T}{(a}_{\left( i \right)}-\tau_{H}) \right]$ and the annual transmission potential (Eq 2, main text), where $\delta_{H0}$ is influential. Conversely, high biting rates are associated with high annual transmission potentials and consequently a low proportion of parasites establishing in the human host, i.e. towards the right of the relationship between $\Pi_{H\left( i \right)}\left[ ATP\left( t-\tau_{H} \right),\Omega_{T}{(a}_{\left( i \right)}-\tau_{H}) \right]$and the annual transmission potential, where $\delta_{H\infty}$ is of higher importance than $\delta_{H0}$.


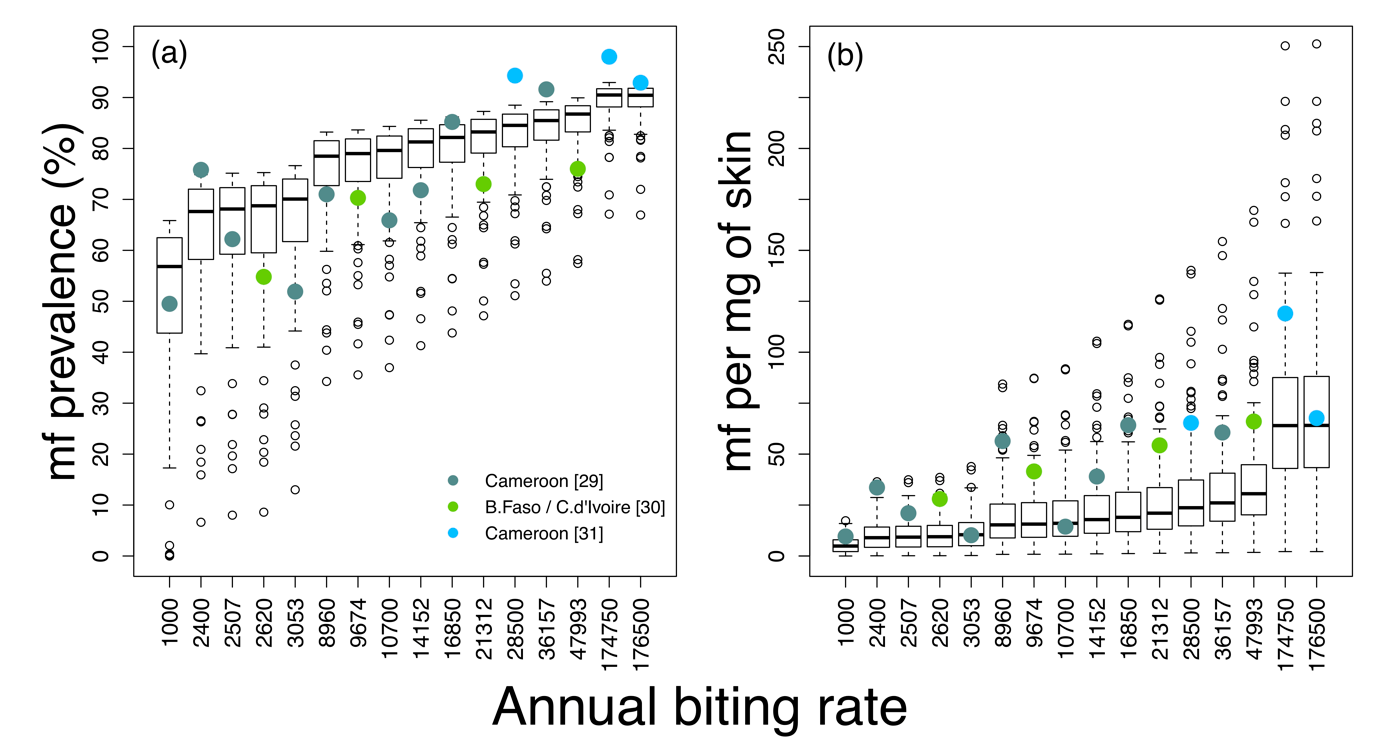


**Figure E. Box and whisker plots of (a) microfilarial prevalence (%) and (b) intensity (microfilariae/mg) predicted by EPIONCHO-IBM for all values of** $\boldsymbol{\delta}_{\boldsymbol{H}\boldsymbol{0}}$**,** $\boldsymbol{\delta}_{\boldsymbol{H\infty}}$ **and** $\boldsymbol{c}_{\boldsymbol{H}}$ **sampled from the Latin hypercube, for exposure parameter *k_E_* = 0.3.** The horizontal black lines are the median, the upper and lower limits of the box are the 75^th^ and 25^th^ percentiles, the whiskers extend 1.5 times the interquartile range from the box, and the open circles are outliers. The solid coloured points represent the data used for fitting.


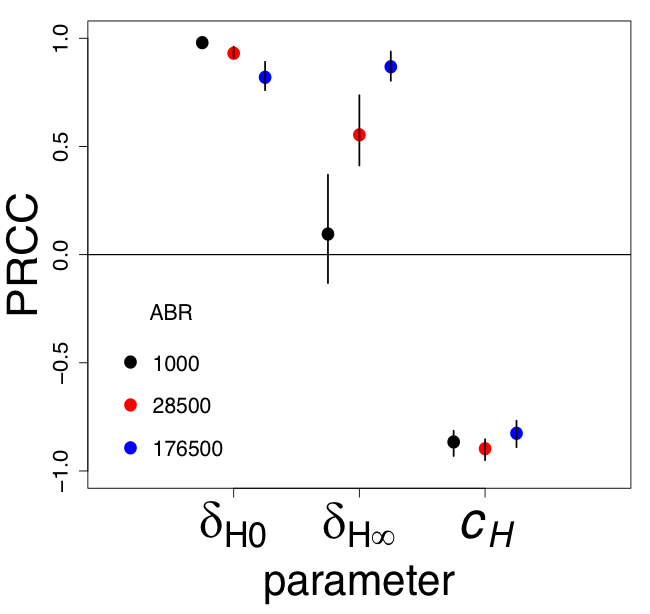


**Figure F. Partial rank correlation coefficients (PRCC) for each within-humans density-dependent parasite establishment parameter set for *k_E_* = 0.3 for three annual biting rates taken from the range used for fitting the model to data.** Confidence intervals were obtained using 1000 bootstrap samples.

To attempt to understand the relative contributions of exposure heterogeneity and density dependence on prevalence dynamics under treatment, we fixed one process whilst altering the strength of the other. Firstly, we set $k_{E}$ = 0.3 and used the density dependence parameters estimated for $k_{E}$ = 0.2 and $k_{E}$ = 0.4, to compare how the severity of density dependence in parasite establishment within humans influences the dynamics. As expected, stronger density dependence (i.e. the parameters estimated for $k_{E}$ = 0.2, Fig G, red line) generated more resilience during treatment than weaker density dependence (i.e. the parameters estimated for $k_{E}$ = 0.4, Fig G, blue line). Greater severity of density dependence was not only reflected by the value of $c_{H}$which was double than with weaker density dependence (0.008 vs. 0.004), but also, an initial proportion of establishing parasites, $\delta_{H0}$, that was three times as large (0.385 vs. 0.118). Therefore, to achieve the baseline microfilarial prevalence used in Fig G (72%), a higher annual biting rate was required for the weaker density dependence parameter set (ABR = 4500) than for the stronger density dependence parameters (ABR = 1400). Second, and for the density dependence parameters estimated with $k_{E}$ = 0.3, exposure heterogeneity was varied. Stronger heterogeneity ($k_{E}$ = 0.2) resulted in more resilience during treatment (Fig H, red line) than when exposure heterogeneity was weaker ($k_{E}$ = 0.4) (Fig H, blue line). In contrast to the analysis varying the severity of density dependence, increasing the strength of exposure heterogeneity (and thus not altering the values of $\delta_{H0}$, $\delta_{H\infty}$,$c_{H}$) resulted in a higher annual biting rate (5000 compared to 800) being required to reach the 64% microfilarial prevalence used in Fig H. (The values of baseline prevalence shown in Figs G and H were chosen to illustrate best the relative contributions of density dependence and exposure heterogeneity to treatment dynamics.) Higher exposure heterogeneity for a given prevalence and (nominal) values of the density dependence parameter set, produces more resilience during treatment partly due to a higher annual biting rate, whereas altering the values of the density dependence parameter set (e.g. increasing the initial proportion of establishing adult worms) and fixing exposure heterogeneity allows a lower annual biting rate to achieve a given prevalence, with increases in resilience not being due to changes in the annual biting rate (when comparing to the weaker density dependence parameter set). Obviously, these parameter sets are derived by fitting the model to the epidemiological data rather than informed by experimental immunological observations which, as discussed in the main text, is a key uncertainty in the model which calls for empirical data to be obtained. Omitting density dependence completely and setting the proportion of parasites establishing within humans to its minimum value, $\delta_{H\infty}$= 0.003, notably decreased resilience to treatment and caused the prevalence to decrease faster under treatment but did not permit to model low (hypoendemic) microfilarial prevalence values. Removing density dependence also led to higher threshold biting rates (in the thousands rather than in the hundreds), not compatible with values recorded in the field [19] (results not shown).

**Figure G. Microfilarial prevalence dynamics under ivermectin treatment assuming** $\boldsymbol{k}_{\boldsymbol{E}}$ **= 0.3 and two different strengths of density dependence (DD) in parasite establishment within humans.** The level of exposure heterogeneity was fixed ($k_{E}$ = 0.3) and the parameters of DD that corresponded to $k_{E}$ = 0.2 (more severe DD, red line) and to $k_{E}$ = 0.4 (weaker DD, blue line) were used to simulate the dynamics of microfilarial prevalence with 25 years of annual ivermectin treatment (80% therapeutic coverage and 1% of systematic non-adherence). Annual biting rates necessary to model the 72% baseline prevalence were 1400 bites/person/year for stronger density dependence and 4500 for weaker density dependence.

**Figure H. Microfilarial prevalence dynamics under ivermectin treatment for two levels of exposure heterogeneity,** $\boldsymbol{k}_{\boldsymbol{E}}$ **= 0.2 and** $\boldsymbol{k}_{\boldsymbol{E}}$ **= 0.4. The same parameter values for density dependent establishment of adult worms within humans (those estimated for** $\boldsymbol{k}_{\boldsymbol{E}}$ **= 0.3) were used for both lines.** The level of exposure heterogeneity was varied (blue line for weaker overdispersion; red line for stronger overdispersion) and the parameters for density dependence that corresponded to $k_{E}$ = 0.3 were used to simulate the dynamics of microfilarial prevalence with 25 years of annual ivermectin treatment (80% therapeutic coverage and 1% of systematic non-adherence). Annual biting rates necessary to model the 64% baseline prevalence were 5000 bites/person/year for $k_{E}$ = 0.2 and 800 for $k_{E}$ = 0.4.

**Supplementary references**

1. Basáñez MG, Boussinesq M. Population biology of human onchocerciasis. Philos Trans R Soc Lond B Biol Sci. 1999;354(1384): 809–826.

2. Basáñez MG, Walker M, Turner HC, Coffeng LE, de Vlas SJ, Stolk WA. River blindness: mathematical models for control and elimination. Adv Parasitol. 2016;94:247–341.

3. Imperial College Research Computing Service,  DOI: [10.14469/hpc/2232](http://doi.org/10.14469/hpc/2232)

4. Prost A, Gorim de Ponsay. [The epidemiological significance of neo-natal parasitism with microfilariae of *Onchocerca volvulus* (authors’ translation)]. Tropenmed Parasitol. 1979;30(4): 477–481 [In French].

5. Filipe JAN, Boussinesq M, Renz A, Collins RC, Vivas-Martinez S, Grillet ME, Little MP, Basáñez MG. Human infection patterns and heterogeneous exposure in river blindness. Proc Nat Acad Sci U S A. 2005;102(42):15265–15270.

6. Basáñez MG, Collins RC, Porter CH, Little MP, Brandling-Bennett D. Transmission intensity and the patterns of *Onchocerca volvulus* infection in human communities. Am J Trop Med Hyg. 2002;67(6):669–679.

7. Dietz K. The population dynamics of onchocerciasis. In: Anderson RM, editor. Population Dynamics of Infectious Diseases. London: Chapman and Hall; 1982. pp. 209–241.

8. Schulz-Key H, Karam M. Periodic reproduction of *Onchocerca volvulus*. Parasitol Today. 1986;2(10):284–286.

9. Lamberton PHL, Cheke RA, Walker M, Winskill P, Crainey JL, Boakye DA, Osei-Atweneboana MY, Tirados I, Wilson MD, Tetteh-Kumah A, Otoo S, Post RJ, Basañez MG. Onchocerciasis transmission in Ghana: the human blood index of sibling species of the *Simulium damnosum* complex. Parasit Vectors. 2016;9(1):432.

10. Prost A. Latence parasitaire dans l'onchocercose. Bull World Health Organ. 1980;58(6): 923–925.

11. Karam M, Schulz-Key H, Remme J. Population dynamics of *Onchocerca volvulus* after 7 to 8 years of vector control in West Africa. Acta Trop. 1987;44(4):445–457.

12. Duke BOL. The effects of drugs on *Onchocerca volvulus* I. Methods of assessment, population dynamics of the parasite and the effects of diethylcarbamazine. Bull World Health Organ. 1968;39(2):137–146.

13. Basáñez MG, Pion SDS, Boakes E, Filipe JAN, Churcher TS, Boussinesq M. Effect of single-dose ivermectin on *Onchocerca volvulus*: a systematic review and meta-analysis. Lancet Infect Dis. 2008;8(5):310–322.

14. May RM. Togetherness among schistosomes: its effects on the dynamics of the infection. Math Biosci. 1977;35:301–343.

15. Schulz-Key H. Observations on the reproductive biology of *Onchocerca volvulus*. Acta Leiden. 1990;59(1-2):27–44.

16. Plaisier AP, van Oortmarssen GJ, Remme J, Habbema JDF. The reproductive lifespan of *Onchocerca volvulus* in West African savanna. Acta Trop. 1991;48(4):271–284.

17. Remme J, Ba O, Dadzie KY, Karam M. A force-of-infection model for onchocerciasis and its applications in the epidemiological evaluation of the Onchocerciasis Control Programme in the Volta River basin area. Bull World Health Organ. 1986;64(5):667-81.

18. Basáñez MG, Razali K, Renz A, Kelly D. Density-dependent host choice by disease vectors: epidemiological implications of the ideal free distribution. Trans R Soc Trop Med Hyg. 2007;101(3):256–269.

19. Walker M, Stolk WA, Dixon MA, Bottomley C, Diawara L, Traoré MO, de Vlas SJ, Basáñez MG. Modelling the elimination of river blindness using long-term epidemiological and programmatic data from Mali and Senegal. Epidemics. 2017;18: 4–15.

20. Eichner M. *Onchocerca volvulus* (Nematoda, Filarioidea) und *Simulium damnosum*-Komplex (Diptera): Die Entwicklung intrathorakal injizierter Mikrofilarien in verschiedenen Überträgerspecies Kameruns. Diplomarbeit, Universität Tübingen, Fakultät für Biologie, Germany. 1989. <http://epimos.com/index.php?id=141&L=1>.

21. Basáñez MG, Churcher TS, Grillet ME. *Onchocerca-Simulium* interactions and the population and evolutionary biology of *Onchocerca volvulus*. Adv Parasitol. 2009;68: 263–313.

22. Cheke RA, Basáñez MG, Perry M, White MT, Garms R, Obuobie E, Lamberton PHL, Young S, Osei-Atweneboana MY, Intsiful J, Shen M, Boakye DA, Wilson MD. Potential effects of warmer worms and vectors on onchocerciasis transmission in West Africa. Philos Trans R Soc Lond B. 2015;370(1665). pii: 20130559.

23. Routledge I, Walker M, Cheke RA, Bhatt S, Nkot PB, Matthews GA, et al. Modelling the impact of larviciding on the population dynamics and biting rates of *Simulium damnosum* (s.l.): implications for vector control as a complementary strategy for onchocerciasis elimination in Africa. Parasit Vectors. 2018;11(1):316.

24. Plaisier AP, Alley ES, Boatin BA, van Oortmarssen GJ, Remme J, de Vlas SJ, Bonneux L, Habbema JDF. Irreversible effects of ivermectin on adult parasites in onchocerciasis patients in the Onchocerciasis Control Programme in West Africa. J Infect Dis. 1995; 172(1):204–210.

25. Kershaw WE, Duke BOL, Budden FH. Distribution of microfilariae of *O. volvulus* in the skin; its relation to the skin changes and to eye lesions and blindness. Br Med J. 1954;2(4890):724–729.

26. Moreau JP, Prost A, Prod'hon J. [An attempt to normalize the methodology of clinico parasitologic surveys of onchocerciasis in West-Africa (authors translation)]. Med Trop (Mars). 1978;38(1):43–51 [In French].

27. Bottomley C, Isham V, Vivas-Martínez S, Kuesel AC, Attah SK, Opoku NO, Lustigman S, Walker M, Basáñez MG. Modelling Neglected Tropical Diseases diagnostics: the sensitivity of skin snips for *Onchocerca volvulus* in near elimination and surveillance settings. Parasit Vectors. 2016;9(1):343.

28. Picq JJ, Jardel JP. [A method of evaluating microfilaria densities of *Onchocerca volvulus* Leuckart, 1893, in onchoceriasis patients. Assessment of microfilarial densities by site and levels of prevalence in skin biopsies; variations of microfilarial densities over a 24 hour period]. Bull World Health Organ. 1974;51(2):145–153 [In French].

29. Renz A, Wenk P. Studies on the dynamics of transmission of onchocerciasis in a Sudan-savanna area of North Cameroon I. Prevailing *Simulium* vectors, their biting rates and age-composition at different distances from their breeding sites. Ann Trop Med Parasitol. 1987;81(3):215–228.

30. Thylefors B, Philippon B, Prost A. Transmission potentials of *Onchocerca volvulus* and the associated intensity of onchocerciasis in a Sudan-savanna area. Tropenmed Parasitol 1978;29(3):346–354.

31. Duke BOL, Anderson J, Fuglsang H. The *Onchocerca volvulus* transmission potentials and associated patterns of onchocerciasis at four Cameroon Sudan-savanna villages. Tropenmed Parasitol. 1975;26(2):143–154.

32. Wu J, Dhingra R, Gambhir M, Remais JV. Sensitivity analysis of infectious disease models: methods, advances and their application. J R Soc Interface. 2013;10(86):20121018.
